# Dynamics of Starter and Non-Starter Lactic Acid Bacteria Populations in Long-Ripened Cheddar Cheese Using Propidium Monoazide (PMA) Treatment

**DOI:** 10.3390/microorganisms10081669

**Published:** 2022-08-19

**Authors:** Zoha Barzideh, Myra Siddiqi, Hassan Mahmoud Mohamed, Gisèle LaPointe

**Affiliations:** 1 Department of Food Science, University of Guelph, Guelph, ON N1G 2W1, Canada; 2 Faculty of Computer and Artificial Intelligence, Benha University, Banha 13518, Egypt

**Keywords:** cheese, microbiota, viable bacteria, propidium monoazide, metagenetics

## Abstract

The microbial community of industrially produced Canadian Cheddar cheese was examined from curd to ripened cheese at 30–32 months using a combination of viable plate counts of SLAB (GM17) and NSLAB (MRSv), qPCR and 16S rRNA gene amplicon sequencing. Cell treatment with propidium monoazide excluded DNA of permeable cells from amplification. The proportion of permeable cells of both *Lactococcus* spp. and *Lacticaseibacillus* spp. was highest at 3–6 months. While most remaining *Lacticaseibacillus* spp. cells were intact during later ripening stages, a consistent population of permeable *Lactococcus* spp. cells was maintained over the 32-month period. While *Lactococcus* sequence variants were significant biomarkers for viable cheese curd communities at 0–1 m, *Lacticaseibacillus* was identified as a distinctive biomarker for cheeses from 7 to 20 months. From 24 to 32 months, *Lacticaseibacillus* was replaced in significance by four genera (*Pediococcus* and *Latilactobacillus* at 24 m and at 30–32 m, *Secundilactobacillus* and *Paucilactobacillus*). These results underscore the importance of monitoring potential defects in cheeses aged over 24 months, which could be diagnosed early through microbial DNA profiling to minimize potential waste of product. Future perspectives include correlating volatile flavor compounds with microbial community composition as well as the investigation of intra-species diversity.

## 1. Introduction

Cheddar cheese forms a significant proportion of the international cheese trade and is produced in many countries today, mostly from cow milk but also using milk from other species such as buffalo. Cheddar cheese production in Canada has risen by 27% over the past 15 years, from 132,000 metric tons in 2005 to 168,067 metric tons in 2021 [1], of which 30% was produced in facilities located in the province of Ontario. This increased production poses a challenge to accurately match the cheese characteristics to consumer preferences and demand, while avoiding economic losses due to cheese defects.

Cheddar can be described as a semi-hard to hard rennet-coagulated cow’s milk cheese that is classified according to ripening indices (from 2–4 months to 6 years) from internal bacterial ripening. Although the name comes from the origin of the cheese in the village of Cheddar (England), the term has become associated with the specific process of “cheddaring”. In the traditional manner, cheddaring involves cutting drained matted and heated curds into slabs which would be stacked, doubled over and re-stacked to express whey out of the cheese [2]. This practice would ensure adequate acid development and keep heat within the curd. In current industrial facilities, the curd is piled in automated systems based on a tower or on a conveyor belt. After acidity development, the curd is milled and dry-salted, which halts the starter acidification activity. Curds are then pressed into blocks and ripened at 4–8 °C for a range of 4 months to over 2 years, depending on the characteristics desired. Thus, the difficulty in manufacturing Cheddar cheese lies in the long ripening period to achieve mature flavor, which can deviate by the formation of off-flavors and textural defects such as slits leading to lower quality cheese [2]. Cheddar has a smooth texture when young, evolving to a slightly crumbly texture and sharp taste when ripened for longer periods.

Much research has been invested in defining the culturable microbiota of Cheddar cheese, and more recently to test the ability of bacterial species to cause defects such as discoloration [3], off-flavors, or slit openings [4]. However, without extensive collection of isolates from multiple types of media or experimental introduction of specific species, mainly the dominant species are revealed. These can be categorized into three groups: the starter lactic acid bacteria (SLAB), adjunct lactic acid bacteria (ALAB), and non-starter lactic acid bacteria (NSLAB).

The SLAB are lactose fermenting strains of *Lactococcus lactis,* producing lactic acid from lactose [5,6], thus contributing to cheese texture through curd acidification [5]. The second group composed of the ALAB acts as ripening aids that may be added to the milk before coagulation. The third group consisting of the NSLAB [7,8] are predominantly comprised of lactobacilli, pediococci, enterococci, and leuconostoc [6]. Proteolysis by both SLAB and NSLAB contribute to developing the texture and flavor of maturing Cheddar [9]. Both SLAB and NSLAB provide peptidase enzymes responsible for converting peptides to amino acids. During ripening, starter lactococci can further breakdown amino acids into flavor compounds [10]. Although lactococci and lactobacilli have shown weak lipolytic activity, both SLAB and NSLAB can contribute to developing free fatty acids in Cheddar cheese (as reviewed by Collins et al. [11]).

The limitations of culture-dependent methods when applied to profiling the succession of bacteria in cheese include the underestimation of the number of species, with the advantage of providing data on the viable population of microbes, depending on the type of media used, which may be more or less specific [12]. According to the standard Cheddar cheese ripening model from culture-dependent methods, the initially dominant starter lactococci are gradually superseded by lactobacilli, either from naturally present adventitious bacteria, or from intentionally added adjunct cultures [13]. Other studies have shown that some lactococci can survive much longer than previously thought [14]. Flow cytometry has revealed that lactococci progress from a viable reproducing state to permeable non-reproducing cells, while autolytic strains will lyse, consequently freeing their cell contents into the cheese environment [15]. Thus, the rate of decline of lactococci depends on the autolytic capacity of the mixture of starter strains used, along with their tolerance to the cooking temperature, salt concentration, and pH developed in the cheese.

Culture-independent techniques were essentially developed to address the limitations of culture media, expanding the characterization of microbial biodiversity while defining the dynamics of SLAB and NSLAB microbiota in cheese during ripening as well as investigating the effects of heat treatment of the milk, spatial location, climate and seasonal effects [16]. Metagenetics uses an amplicon-based high throughput sequencing approach, which target genes of taxonomic significance that have been used to study the microbial communities of starters and dairy products [17,18,19]. The standard 16S rRNA gene amplicon sequencing method (metabarcoding) does not discriminate between viable and dead bacteria, as it is based on DNA extraction from all cells, whether dead or viable. In comparing Cheddar cheese of increasing age from mild (up to 6 months) to vintage (up to 32 months), Ashfari et al. [20] revealed that three brands of cheese did not show the same changes in the microbiota composition over time when analyzed using 16S rRNA gene sequencing. While *Lactococcus* DNA dominated throughout ripening for Brand A, *Lactobacillus* DNA became predominant by 18 months (Extra tasty grade) for Brand C. Brand B showed a completely different profile with *Streptococcus* DNA dominating up to 18 months, then *Lactococcus* dominating the Vintage cheese at 32 months (very little *Lactobacillus*). Long-ripened Cheddar cheese may thus contain a highly variable accumulation of *Lactobacillus* among brands, which would differentiate the flavor profile of the respective products. Better knowledge of the effect of these changes on the finished product profile will help the cheese industry to design the optimal ripening time and community composition to target specific consumer niches and reduce waste.

Analyzing total DNA is helpful in monitoring the bacterial history of the cheese, but it does not permit discrimination between viable and dead or non-growing microorganisms. This distinction will help to predict the microbes contributing to metabolic activity during later ripening stages. Propidium monoazide (PMA) can be used for the amplification of the viable bacteria in cheese. PMA is a DNA-intercalating dye that can bind free DNA or diffuse into cells with compromised membranes. Once inside the cells, PMA intercalates into the DNA and can be covalently cross-linked by exposure to light, which then strongly inhibits PCR amplification of DNA from these permeable cells [21]. Therefore, treatment of the cells with PMA before DNA extraction hinders the subsequent amplification of the DNA by PCR, which provides a profile of the presumably viable bacteria with intact cell membranes [17]. A reduction in PMA-treated DNA compared to total DNA would mean more permeable cells in a sample and a lower number of intact viable cells. Even though membrane integrity is not proof for cell activity, identifying the intact cells would be a step forward in a more comprehensive view of the total bacterial community. This method has been validated for a number of bacterial species, including *Lactococcus lactis* [17] and *Lactobacillus* [22,23] both in pure culture and in a cheese matrix [4].

Erkus et al. [17] used the whole genome sequencing approach to monitor the microbial viability of both the undefined starter cultures and microbial communities during Gouda cheese ripening. They were able to quantify the relative abundance of seven genetic lineages of *L. lactis* as well as one lineage of *Leuconostoc mesenteroides* subsp. *cremoris* from lineage-specific biomarker sequences in complex starter cultures and cheese. They found that PMA treatment was not a limitation to sequencing and its use was able to provide selective profiling for viable populations in a Gouda cheese microbial community. The relative contribution of specific genetic lineages was shown to differ between PMA-treated and non-PMA-treated samples, suggesting that some lineages remained viable longer than others in Gouda cheese [17]. Similarly, O’Sullivan et al. [24] studied the microbial communities of continental-type cheese using 16S rRNA gene amplicon sequencing, revealing spatial (rind vs core) and temporal variations (early versus late production day manufacturing) in population composition, as well as the influence of abiotic factors. Core microbiota were more diverse than the rind up to 64 days of ripening, with salt tolerant microbes, then aerobic and aerotolerant genera prevalent on the rind. In the core, *Clostridium* increased over ripening to attain around 3% relative abundance, although no defects associated with gas production were noted in this case.

Using PMA 16S rRNA gene amplicon sequencing, Xue et al. [4] showed that up to 4 months of ripening; viable nonstarter bacteria were predominantly *Lactobacillus* spp. (44.3 ± 31.1%), *Streptococcus* spp. (29.2% ± 15.8%), and *Staphylococcus* spp. (10.4 ± 9.8%). Only *Lactobacillus* spp. increased over the 120 days of aging whereas *Streptococcus* spp. declined over time, while *Staphylococcus* spp. populations remained constant. At 90 and 120 days of ripening, when slits were found, *Lactobacillus* and *Turicibacter* had increased in relative abundance (averages of 1.2- and 2.2-fold at 90 and 120 days, respectively). *Limosilactobacillus fermentum* was the dominant species of *Lactobacillus* amplicon sequence variant that increased on average 1.1-fold at 120 days. Cheese containing the *L. fermentum* isolates exhibited the highest levels of slit damage. They also showed that the pre- and post-HTST (High-Temperature Short Time)-pasteurization milk used to make the cheese blocks that developed slits could be differentiated by the microbial composition (diversity of lactobacilli, for example) compared to milk that resulted in good-quality cheese without slits.

Given the gap in knowledge about the microbial succession over long ripening periods over 2 years, the aim of our study was to evaluate the microbial diversity and succession in long-ripened Cheddar cheese manufactured at one industrial cheese plant in Ontario over a three-year period using culture-dependent techniques, high throughput 16S rRNA gene amplicon sequencing, and qPCR to quantify target groups of species.

## 2. Materials and Methods

### 2.1. Cheese Manufacturing and Sampling

Cheddar cheeses were produced in an industrial facility using heat-treated (thermized) milk following conventional cheese-making methods; curd was cooked at 38.5 °C, pitched at pH 6.15, milled at pH 5.35, and salted at 2.7% (*w*/*w*). The approximate weight of milk in each vat was 23,000 kg. The same milk was used in six vats per trial using two different starters (three vats per starter) per trial with a rotation of a total of ten starter mixes consisting of *Lactococcus cremoris*. Each starter was repeated on average two or three times over the 13 trials. For each of the 13 trials, six cheese blocks were sampled per time point, each from one of the six separate vats, totaling 132 cheeses. Sampling was performed over a course of 32 m at 0–1 m (13 trials), 3–6 m (13 trials), 7–10 m (13 trials), 13–15 m (13 trials), 18–20 m (11 trials), 24 m (7 trials), and 30–32 m (5 trials) time points. Samples that were received at day zero were in the form of cheese curd, at 1 m, they were cheese plugs and samples that were received at three months and older were blocks of 9 kg (20 lb) cuts that were obtained from the 290 kg (640 lb) mother block. The 9 kg block was cut into half in a way that every 4.5 kg (10 lb) block had two or three exterior and interior faces. Sections of the 4.5 kg block (including internal and external faces) were ground before homogenization in buffer as described below.

### 2.2. Preparing Cheese Homogenate for Bacterial Enumeration and DNA Extraction

Ten grams of each cheese sample was suspended in 90 mL of 45 °C sterile 2% (*w*/*v*) trisodium citrate dehydrate solution and homogenized in stomacher bags (Fisher Scientific, Mississauga, ON, Canada) using Seward Stomacher® 400 Circulator for 5 min at 260 rpm. The resulting homogenate was used for bacterial enumeration and DNA extraction.

### 2.3. Bacterial Enumeration

One milliliter of cheese homogenate was serially diluted in 9 mL of 0.1% peptone water and was plated on selective media for enumeration and cultivation of SLAB and NSLAB. To select for SLAB (mostly *Lactococcus* spp., but other species such as *Enterococcus* spp. may also grow), M17 agar (Oxoid Microbiology, Nepean, ON, Canada) with 0.5% *w*/*v* glucose (Fisher Scientific, Mississauga, ON, Canada) hereafter referred to as GM17 was used following incubation at 30 °C for 72 h. De Man, Rogosa and Sharpe (MRS) agar (Oxoid Microbiology, Nepean, ON, Canada) with 1 mg/L of vancomycin (Sigma-Aldrich, St. Louis, USA) hereafter referred to as MRSv was used to select for mesophilic NSLAB, which was incubated under anaerobic conditions in a GasPak jar at 30 °C for 72 h.

### 2.4. Propidium Monoazide (PMA) Treatment

PMA treatment of cells was performed following the protocol described by Desfossés-Foucault et al. [23] with some modifications. Each bacterial pellet obtained as described below in Section 2.5 was suspended in 500 μL of 0.1% buffered peptone water and treated with 5 μL of 2.5 mM PMA solution (PMAxx dye, Biotium, Fermont, USA) diluted in nuclease-free water. Samples were incubated on ice in the dark for 15 min. Samples were then exposed to PMA UV light (PhAST Blue, GenIUL, Barcelona, Spain) for 15 min and placed on ice in the dark for another 15 min. The PMA-treated cell suspensions were centrifuged at 10,000× *g* for 5 min, washing the cell pellets with 500 μL of 2% *w*/*v* sodium citrate solution followed by centrifugation at 10,000× *g* for 1 min and discarding the supernatant. PMA-treated cell pellets were used for DNA extraction.

### 2.5. DNA Extraction from Cheese Samples

Ten milliliter of the cheese homogenate was centrifuged at 10,000× *g* for 10 min at room temperature. After centrifugation, the supernatant was removed, and the fat layer was cleared as much as possible with a sterile cotton swab. Once the fat layer was cleared, cells were suspended in 1 mL of 2% sodium citrate solution for washing. The cell suspension was centrifuged at 10,000× *g* for 5 min and the supernatant was removed. This step was repeated 3–4 times until no fat was left in the tube. The resulting cell pellet was used for DNA extraction for determining the total and viable bacterial composition. DNA extraction was carried out on cell pellets with and without PMA treatment using Invitrogen PureLink Microbial DNA Purification kit following the manufacturer’s instructions (Invitrogen Canada Inc., Burlington, ON, Canada) with some modifications.

Bacterial pellets were suspended in 800 μL of lysis buffer and transferred to a 2-mL microtube containing 0.3 g of zirconium beads (1-mm diameter). One hundred microliter of lysis enhancer was added, vortexed briefly, and incubated at 75 °C for 10 min. Tubes were shaken for 10 min at maximum speed on the vortex mixer and were then centrifuged at 14,000× *g* for 2 min. Up to 500 μL of the supernatant was transferred to a clean microcentrifuge tube, avoiding the bead pellet and any debris. Nine hundred microliter of binding buffer was added, vortexed briefly, loaded onto a spin column-tube assembly and centrifuged at 14,000× *g* for 1 min. The column was washed using 500 μL of wash buffer, and DNA was eluted using 50 μL of elution buffer followed by storage at –20 °C until further analysis. For cheese samples with lower cell count, mostly samples older than one year, three to six cell pellets were treated separately up to the step that the DNA was loaded on the DNA binding column. The extracted nucleic acids from all cell pellets were loaded on a single column, washed, and collected as one sample.

### 2.6. Quantification of Bacterial Species by qPCR

Absolute quantification of target *Lactococcus* spp., *Lactobacillus* spp., and *L. casei/paracasei* was performed on total and PMA-treated DNA. Quantitative PCR was carried out in 96-well plates (Hard-Shell Thin-Wall Skirted PCR Plates, Bio-Rad, Mississauga, ON, Canada) using a Bio-Rad real time CFX96 system. Each 20 μL amplification was done in triplicate and contained 10 μL of SsoAdvanced Universal Inhibitor tolerant SYBR Green Supermix (BioRad), 3 μL of RNase free water, 5 μL of DNA, and 1 μL of both forward and reverse 10 μM primer (final concentration of 0.5 μM). A no-template control (NTC) was included on each plate, which had 5 μL of RNase free water instead of the DNA template, as well as a negative control. Amplification was started with denaturation at 95 °C for 10 min, followed by 40 cycles of 95 °C for 30 s and 60 °C (for *Lactococcus* spp. and *L. casei/paracasei*) and 61.5 °C (for *Lactobacillus* spp.) for 30 s (Table 1). After amplification, a dissociation curve analysis was performed by increasing the temperature by 0.5 °C every second from 65 to 95 °C to confirm the absence of non-specific amplification products. A 133-bp and 123-bp region in the V3 region of the 16S rRNA gene was targeted for *Lactococcus* spp. and *L. casei/paracasei,* respectively (Table 1). For *Lactobacillus* spp., the forward primer targets the 16S/23S rRNA intergenic spacer region while the reverse primer is specific to the terminal region of the 16S rRNA gene (Table 1).

For the DNA standard curve, DNA from *Lactococcus lactis* subsp. *cremoris* strain LLG1000 (for *Lactococcus* spp.) and *L. paracasei* strain LLG2134 (for *L. casei/paracasei* and *Lactobacillus* spp.) was extracted using DNeasy Blood and Tissue DNA extraction kit (Qiagen) according to the manufacturer’s instructions with some modifications (centrifugation at 10,000× *g*; cell pellet suspended in 200 μL lysis buffer, 62.5 μL proteinase K added instead of 25 μL, column washed with 200 μL instead of 500 μL AW1 and AW2, incubations for 1 h instead of 30 m, DNA elution in 50 μL instead of 200 μL). Both strains were isolated from the cheeses in this study and identification was confirmed by MALDI-TOF. DNA quality and concentration were measured using a Nanodrop spectrophotometer (NanoDrop 1000 spectrophotometer, Thermo Scientific, Burlington, ON, Canada) and a Qubit™ 4 Fluorometer with the QuBit® dsDNA BR assay kit (Invitrogen Canada Inc., Burlington, ON, Canada). A serial dilution of 1 in 10 concentrations of each DNA species was prepared up to seven dilutions.

Standard curves were used for conversion of Ct values to gene copy numbers per μL. The target concentration (copies/μL) was calculated from the DNA concentration (cDNA, ng/μL), the Avogadro constant (NA, 6.022 × 10^23^ molecules/mol), the genome size (lDNA, bp), and the average molecular mass of a double-stranded DNA base pair (660 × 10^9^ ng/mol) using the following equation: number of copies per microliter = (cDNA × NA)/(lDNA × 660 × 10^9^) [25]. Results were then expressed as genome copy number/g of cheese.

*Lactococcus*-specific primers were tested for specificity using *L. lactis* subsp. *cremoris* SK11, *L. lactis* subsp. *lactis* IL1403, *Lactococcus lactis* (target) and *L. paracasei* ATCC 334, *L. paracasei*, *L. buchneri*/*L. parabuchneri*, *L. rhamnosus*, *L. brevis*, *L. coryniformis*, *L. plantarum* ATCC 14917, *L. helveticus* ATCC 12046, and *L. zeae* ATCC 15820 (negative control), and *L. casei/paracasei*-specific primers were tested for specificity using *L. paracasei* ATCC 334, *L. paracasei* (target) and *L. lactis* subsp. *cremoris* SK11, *L. lactis* subsp. *lactis* IL1403, *Lactococcus lactis*, *L. buchneri*/*L. parabuchneri*, L. rhamnosus, *L. brevis*, *L. coryniformis*, *L. plantarum* ATCC 14917, *L. helveticus* ATCC 12046, and *L. zeae* ATCC 15820 (as negative control, data not shown). In the current study, we used *Lactococcus lactis* subsp. *cremoris* strain MG1363 as negative control for *L. casei/paracasei* and *Lactobacillus* sp. primers and *L. paracasei* LLG2134 as negative control for *Lactococcus* sp. primers. 

**Table 1:** qPCR primers and standard curve results for 16S rRNA gene quantification of Cheddar cheese bacteria.

Bacterial Species	16S rRNA Primers Sequence (5′ → 3′)	Amplicon Length (bp)	Slope	Intercept Point	Efficiency (%)	R^2^
*Lactococcus* sp. ^1^	^3^ F: GAGGCAGCAGTAGGGAATCTTC R: CTGATGAGCTTT-CCACTCTCA	133	−3.486	33.729	93.6	0.997
*L. casei/paracasei* ^1^	F: GTGCTTGCACTGGATTCGACTTA R: TGCGGTTCTTGGATCTATGCG	123	−3.250	35.928	103.1	0.998
*Lactobacillus* sp. ^2^	F: CTCAAAACTAAA- CAAAGTTTC R: CTTGTACACA-GCCCGTCA	250	−3.494	35.937	93.3	0.992

^1^ Desfossés-Foucault et al. [23]; ^2^ Dubernet et al. [26]. *Lactobacillus* species detected include: *L. acidophilus* CNRZ 204, *L. amylovorus* CIP 102989, *L. delbrueckii* subsp. *bulgaricus* CIP 101027, *L. delbrueckii* subsp. *delbrueckii* CNRZ 225, *L. delbrueckii* subsp. *lactis* CNRZ 207, *Lactobacillus farciminis* CIP 103136, *Lactobacillus gallinaum* CIP 103611, *L. helveticus* CNRZ 223, *L. johnsonii* CIP 103653, *L. ruminis* CIP 103153, *L. casei* subsp. *casei* CNRZ 313, *Lactobacillus coryniformis* subsp. *torquens* CIP 103134, *L*. *graminis* CIP 105164, *L. paracasei* subsp. *paracasei* CNRZ 763, *L. pentosus* CIP 103156, *L. plantarum* CNRZ 211, *L. rhamnosus* CIP A157, *L. zeae* CIP 103253, *Lactobacillus buchneri* CIP 103023, *L. fermentum* CNRZ 209, *L. reuteri* CIP 101887, *Lactobacillus suebicus* CIP 103411. With the revised taxonomy of *Lactobacillus* [27], these primers could bind with DNA from the following genera: *Lactobacillus*, *Lacticaseibacillus*, *Lactiplantibacillus*, *Latilactobacillus*, *Ligilactobacillus*, *Limosilactibacillus*, *Liquorilactobacillus;* ^3^ F: forward primer, R: reverse primer.

### 2.7. Community Profiling Using 16S rRNA Gene Amplicon Sequencing

DNA from all cheese samples was subjected to 16S rRNA gene amplicon sequencing using the MiSeq platform (Illumina, San Diego, CA, USA). For each sample, 10 μL of extracted DNA was adjusted to 5 ng/μL concentration and was sent for amplicon sequencing by the Advanced Analysis Centre genomics facility at the University of Guelph, Guelph, Ontario, Canada. Amplicon sequencing libraries were prepared following the procedures described in the 16S Metagenomics Sequencing Library Preparation Guide with modifications (Illumina 2020). V3 and V4 regions (~460 bp) of the 16S rRNA gene were amplified using the following universal primers: forward primer: 5’ CCTACGGGNGGCWGCAG and reverse primer: 5′GACTACHVGGGTATCTAATCC [28]. The primers for the first stage polymerase chain reaction (PCR) consisted of overhang nucleotide sequences (to ensure compatibility with the Illumina index) and locus-specific sequences.

The first-stage PCR reaction mix (25 μL) contained 1x KAPA HiFi HotStart Ready Mix (KAPA Biosystems), 0.2 μM of each primer (Integrated DNA Technologies), and 5 μL template DNA. The PCR thermal cycling conditions were 95 °C for 3 min; 30 cycles of 95 °C for 30 s, 55 °C for 30 s, and 72 °C for 30 s; followed by 72 °C for 5 min using a GeneAmp PCR System 9700 thermal cycler (Life Technologies, Burlington, ON, Canada). The PCR products were purified using NucleoMag Beads (Macherey-Nagel, D-Mark Biosciences, Toronto, ON, Canada) to removed free primers and primer dimers. The purified PCR products were re-amplified using the Nextera XT Index kit. The indexes were 8 bases long, and each sample for each target was dual indexed.

This second-stage PCR reaction mix (50 μL) contained 1x KAPA HiFi HotStart Ready Mix (KAPA Biosystems), 5 μL each of Nextera XT Index Primers (Illumina), and 5 μL template DNA. The PCR thermal cycling conditions were 95 °C for 3 min; 8 cycles of 95 °C for 30 s, 55 °C for 30 s, and 72 °C for 30 s; followed by 72 °C for 5 min 42 using a GeneAmp PCR System 9700 thermal cycler (Life Technologies). PCR products were purified using NucleoMag Beads (Macherey-Nagel). The purified amplicons were normalized by measuring the concentrations of the purified PCR products using a Qubit, calculating the molar concentrations, and adjusting all molar concentrations to be identical across all samples using a buffer. The purified amplicons were then combined in equal molar ratios based on their DNA concentrations and the pooled fragments were sequenced.

For DNA sequencing using the MiSeq system, the pooled libraries were denatured with NaOH, diluted with hybridization buffer, and then heat-denatured prior to sequencing. PhiX was included at a 15% level to serve as an internal control. Sequencing was conducted using a MiSeq sequencer with the MiSeq v3 reagent kit and 2 × 300 paired-end cycles according to the manufacturer’s protocol. Raw sequence reads were filtered using the MiSeq Sequencer System Software to remove low-quality sequences and trimmed to remove adaptor sequences. The resulting reads were up to 301 bases long.

### 2.8. Data Analysis

All 16S rRNA gene sequence data were analyzed with QIIME2 version 2017.12 (accessed 20 March 2018 at http://qiime2.org) [29] for bacterial taxonomic classification to operational taxonomic units (OTU) to perform diversity analysis. Forward and reverse reads were trimmed at the front ends to remove primer sequences and at the tail ends when quality was consistently below 25. Using DADA2, reads were filtered and combined as OTUs (97%), and chimera were removed. The manually created DAIRYdb reference database was used as a classifier to assign taxonomic names to the representative sequences.

In QIIME2, a rooted tree was constructed to represent sequence diversity that included phylogenetic information (OTU analysis). The QIIME2 diversity “core-metrics-phylogenetic” plugin was used for calculating alpha and beta diversity measures on OTU profiles. For alpha diversity, Chao1 and Shannon indices were selected [14]. For beta diversity, the Bray–Curtis dissimilarity measures were visualized using NMDS [30]. Diversity metrics were compared by grouping samples by starter, trial, and season. The filtered read counts of relative abundance for *Lactococcus* spp., *Lacticaseibacillus, Weissella, Lactobacillus, Streptococcus* genera were compiled from 16S rRNA gene amplicon sequences for cheeses from trials 1 to 13 (% of total reads identified to genus level obtained from OTUs). Moreover, we developed a script using R (V3.6.3) on core i7, 16G RAM, 2.8 GHz processor laptop to visualize the distribution profile of the relative abundance of *Lactococcus* spp., *Lacticaseibacillus* and *Lactobacillus*, *Weissella* and *Streptococcus*, as shown in boxplots for PMA and non-PMA treated trials across seven age groups.

Amplicon sequence variant analysis was carried out on the 16S gene amplicon sequence reads from PMA-treated cheese samples only following the amplicon bioinformatics R script which infers ASVs through dereplication [31]. DAIRYdb was then used as the reference database to classify ASVs to genus level. Non-filtered reads were used to tabulate *Lactococcus* and *Lacticaseibacillus* spp. total ASVs, while filtered ASVs with ≥0.1% relative abundance in ≥10% samples were used to characterize overall biomarkers for cheese ages. The linear discriminant analysis effect size (LEfSe) for microbiome biomarker discovery was carried out on filtered ASV data from all PMA samples using microbiomeMarker R package with a Kruskal–Wallis rank sum test (*p* < 0.05) and an LDA score cutoff of 2.

Statistical analysis on average viable plate counts, relative abundance (genus level), and qPCR was performed using SPSS, version 28.0.1.1 for Mac OS (SPSS statistics, IBM, East Markham, ON, Canada). A Tukey’s honestly significant difference test was used for post-hoc analyses where there were no violations of the test assumptions for the homogeneity of variance (Levene’s k—sample comparison of variances). Age groups that violated these assumptions were subjected to a 1-way ANOVA using the Games–Howell post-hoc analysis. The significance level was set at a probability value of ≤0.05. The independent samples *t* test was used (*p* ≤ 0.05) to compare total and PMA-treated DNA of average of all trials.

## 3. Results

Out of the total of 13 commercial cheese manufacturing trials, cheeses from thirteen trials were ripened to 13–15 m, eleven trials were ripened for 18–20 m, cheeses from seven trials were ripened for 24 m, while five trials were ripened for the full 30–32 m (see Figure S1 in the Supplementary Material for the experimental design).

### 3.1. Culturable SLAB and NSLAB during Ripening

Bacterial dynamics and community composition of culturable bacteria were followed during 30–32 m of ripening over 13 trials. The disodium-β-glycerophosphate in GM17 agar inhibits the growth of many *Lactobacillus* species and therefore is used for SLAB count, which are mainly *Lactococcus* spp., but *Enterococcus* spp., can also grow on this medium. To count the NSLAB, addition of vancomycin to MRS at 1 mg/L inhibits the growth of *Lactococcus* species as well as *Streptococcus thermophilus* [32].

**Figure 1:**
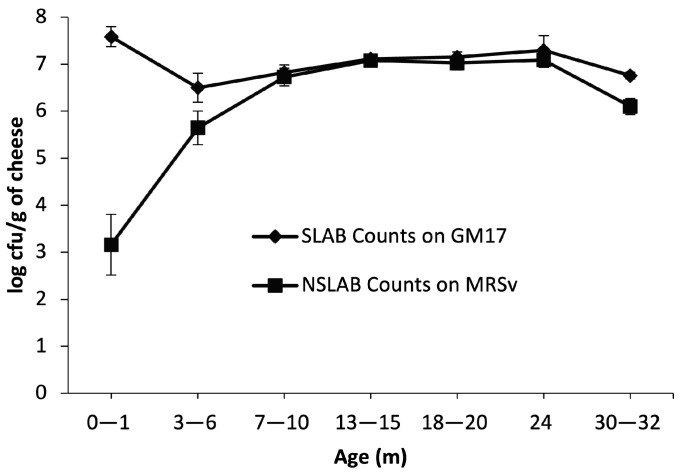
Mean viable counts from cheese plated on GM17 agar (diamond symbols) and MRSv agar (square symbols) to select for SLAB and NSLAB, respectively during 30–32 m of ripening in 13 trials. Values are the means and standard error (no visible bar indicates that SE is smaller than the symbol). Sampling was performed over a course of 32 m at 0–1 m (13 trials), 3–6 m (13 trials), 7–10 m (13 trials), 13–15 m (13 trials), 18–20 m (11 trials), 24 m (7 trials), and 30–32 m (5 trials) time points. Data from individual trials and starters can be found in Supplementary Material Figures S2 and S3.

Culturable counts on GM17 were found to be around 7–9 log at the starting point, with a maximum of 9.9 log cfu/g in trial 13 cheeses. After 3–6 m of ripening, all but 2 trials showed a one to two log decrease (from 7–9 to 5.5–6.5 log cfu/g of cheese). Trial 13 had a significantly higher count at the starting point, which remained almost similar (9.2 log) at 3–6 m and trial 11 showed an increase of 0.5 log from the starting point. The plate count showed another decreasing trend (0.5–1 log) after 24 months, reaching to around 6 to 6.7 log at 30–32 m (Table S1). Variation between trials was even lower at 24 and 30–32 m. At 30–32 m, the counts on GM17 remained as high as 7 log cfu/g, which was close to the starting point (only one log lower) in most trials. From the five trials that were ripened to 30–32 m, only trials 3 and 5 showed a significant decrease between starting and end point (2 log decrease) (Table S1). Overall, average counts on GM17 at the end of ripening were not significantly lower than their starting point in all trials (Figure 1, Table S1). Over ripening, viable counts on GM17 were more often similar between starters used in the same trial than for the same starter in separate trials (Supplementary Material Figure S2). The same starters used In separate trials did not always perform the same in terms of rate of decline over the first 10 months of ripening. Five starters appeared to decline by 1.5 to 2 log cfu/g by 3–6 months in at least one trial, while four starters seemed to take longer to decline (7–10 months).

The culturable NSLAB count at the starting point was around 3 log cfu/g on average, but was below detection level (3 logs) in four out of 13 trials while the 9 other trials ranged from 3 to 6 log cfu/g. By 3–6 m, most trials showed an increase of up to 3.5 log, reaching an average of 5 log cfu/g. From this point to 7–10 m, all trial cheeses (except trial 13) showed an increase of up to 3 logs, reaching 6.5–7 log cfu/g, and stayed around the same value up to 24 m. NSLAB counts in the five trials that were ripened all the way to 30–32 m decreased slightly to around 6–6.5 log cfu/g. The age for attaining the highest value of NSLAB varied from 3–6 m to 24 m time points (7–8 log) depending on the trial and was not necessarily the oldest age for each trial. The variation in NSLAB counts was high between trials at the beginning and up to 3–6 m, where the highest variation was observed with a range of as low as 3.5 to as high as 8 logs. Even though there was a high variation in NSLAB counts among trials at the beginning and up to 3–6 m, trials were closer in the NSLAB viable counts from 7–10 m and after, and at the end of 32 m they were all around the same count at a maximum of 6.5 log (Figure 1).

The ratio of SLAB counts (log cfu/g cheese) plated on GM17 agar to NSLAB plated on MRSv agar for cheeses in 13 trials over 32 m of ripening favored SLAB up to 3–6 m but remained close to 1:1 until the end of ripening, when the ratio again favored viable SLAB over NSLAB.

### 3.2. Quantification of SLAB and NSLAB through qPCR (Total and Viable)

qPCR was carried on DNA extracted from both non-PMA and PMA-treated cells. PMA treatment should prevent amplification of DNA from compromised (permeable) cells, so that amplification represents live and non-permeable cells with intact energized membranes.

**Figure 2:**
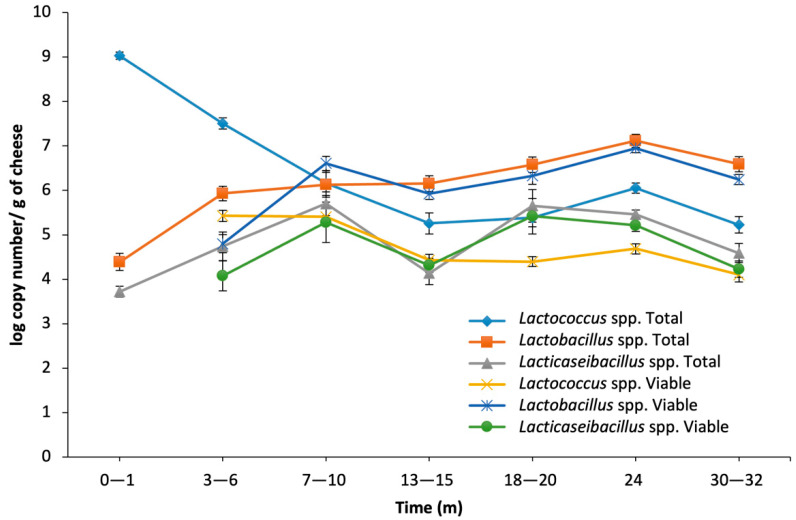
16S rRNA log gene copy number of specific target bacterial genera, *Lactococcus* spp., *Lactobacillus* spp., and *Lacticaseibacillus* spp. (detected in 2 of 5 trials at 0–1 m) per g of cheese measured by qPCR of DNA from total and viable cells (PMA-treated) in cheese samples from five trials ripened to 32 months. Standard error applied to each data point.

*Lactococcus* spp. quantified from total DNA was highest at 0–1 m, decreasing until 30–32 m, where the final numbers were 3–4 log lower than the starting point. The lowest values were also observed at 30–32 m (3.5–4.5 log copy number/g) for PMA-treated DNA, which was 4–5 log lower than the starting point. By 30–32 months, PMA-treated DNA counts were 0.5–2.5 log lower than the total DNA counts at the same time point. PMA-treated DNA showed less fluctuation in gene copy number of *Lactococcus* spp. throughout ripening than the total DNA counts (Figure 2). The biggest difference between total and PMA-treated DNA was 1.5–2.5 log 16S rRNA copy number/g observed at 3–6 m (Figure 2, Supplementary Material Table S2).

*Lactobacillus* spp. qPCR counts were 3.5–5.5 log 16S rRNA gene copy number per g at 0–1 m, which then increased by 1.5 log at 3–6 m (Figure 2). The counts continued to increase slightly up to 24 m, when all trials were around 6–7.5 log. From 24 m to the end point, *Lactobacillus* spp. qPCR counts in most trial cheeses decreased by 0.5 log and reached a final quantity of 6.5–7.5 log. The *Lactobacillus* spp. quantity by total DNA qPCR showed a maximum of 3 log overall increase from cheese curd to 30–32 m (Figure 2). PMA-qPCR for *Lactobacillus* spp. was similar to total DNA over time with slightly lower values for PMA-treated DNA at most time points except 7–10 m. At this time point, intact cells of *Lactobacillus* formed a slightly higher proportion of the population of intact cells than out of the total DNA reads (in terms of relative abundance). The maximum difference between PMA and non-PMA *Lactobacillus* counts was 1–2 log, observed at 3–6 m. At any other time point, this difference was 0–0.5 log and in a few cases up to 1 log. Variation in the *Lactobacillus* spp. copy numbers from trial to trial was higher for total DNA than for PMA-treated DNA (Supplementary Material Table S2).

At 0–1 m, the log copy number of *Lacticaseibacillus* spp. quantified from total DNA and from PMA-treated DNA was below the detection level (2 log) for three out of five trials, so the average value is for the remaining two trials only. At 3–6 m, this value was 3.5–4.5 log and from 3–6 m to 30–32 m it fluctuated between 3 and 8 log 16S rRNA genome copy numbers. The highest value was found at 24 m for four out of five trials (around 5.5 log). At 3–6 m, the increase in PMA-treated *Lacticaseibacillus* spp. DNA ranged widely from 2 to 7 log 16S rRNA gene copy numbers, depending on the trial. From this point to 7–10 m, all five trials showed an increase, reaching 5.5–8.5 log. Although trends varied among trials from 13–15 m to 24 m, all five trials showed a decrease at the 24 to 30–32 m time point, reaching 3–5 log. The highest value was at 24 m for three out of five trials (Figure 2, Supplementary Material Table S2).

The ratio of total *Lactococcus* to *Lactobacillus* was in favor of *Lactococcus* up to 7–10 m and then favored *Lactobacillus* spp. in most trials after that. This switch occurred after 3–6 m for PMA-treated DNA. The initially high ratio of PMA-treated *Lactococcus* spp. over *Lacticaseibacillus* spp. (presumed to be viable cells) at 3–6 months remained closer to 1:1 until 13–15 m (Figure 2), then PMA-treated (viable) *Lacticaseibacillus* exceeded PMA-treated *Lactococcus* until 30–32 m.

### 3.3. Microbial Community Composition Evaluated by 16S rRNA Gene Amplicon Sequences

The filtered read numbers (0.1% abundance in over 10% of the samples) ranged from 89 to 199,518 per sample with an average of 63,215 reads per sample for non-PMA and from 2260 to 141,535 per sample with an average of 62,006 reads per sample for PMA samples. The one sample with the lowest read number (89 reads) was excluded. Based on alpha rarefaction curves, 5000 reads were adequate to capture the diversity of the samples.

Seven main genera were detected in the 713 cheese samples at ≥0.1% relative abundance in ≥10% of samples. A total of 1026 OTUs were detected in non-PMA treated samples (*n* = 376) and a total of 697 in PMA-treated samples (*n* = 337). The bacterial genera found in all samples were *Lactobacillus*, *Lacticaseibacillus, Lactococcus*, *Pediococcus*, *Streptococcus*, *Weissella*, and genera of *Enterobacteriaceae*. The abundance of each genus changed over ripening with *Lactococcus* being the dominant genus at early stages and *Lactobacillus/Lacticaseibacillus* in later ripening periods.

**Figure 3:**
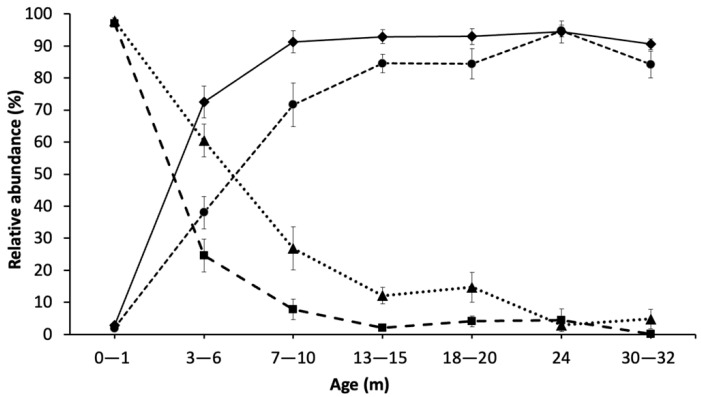
Relative abundance of *Lactococcus* spp. and NSLAB using 16S rRNA gene amplicon sequences for cheeses from 13 trials (% of total reads identified to genus level). Triangle symbol: *Lactococcus* non-PMA-treated total DNA; Square symbol: *Lactococcus* PMA-treated DNA (viable cells); Diamond symbol: NSLAB PMA-treated DNA (viable cells); Circle symbol: NSLAB non-PMA-treated total DNA. The genera compiled as NSLAB were: *Lactobacillus*, *Lacticaseibacillus*, *Paucilactobacillus*, *Loigolactobacillus*, *Lentilactobacillus*, *Secundilactobacillus*, *Weissella*, *Streptococcus*, *Pediococcus*, and *Leuconostoc*.

**Figure 4:**
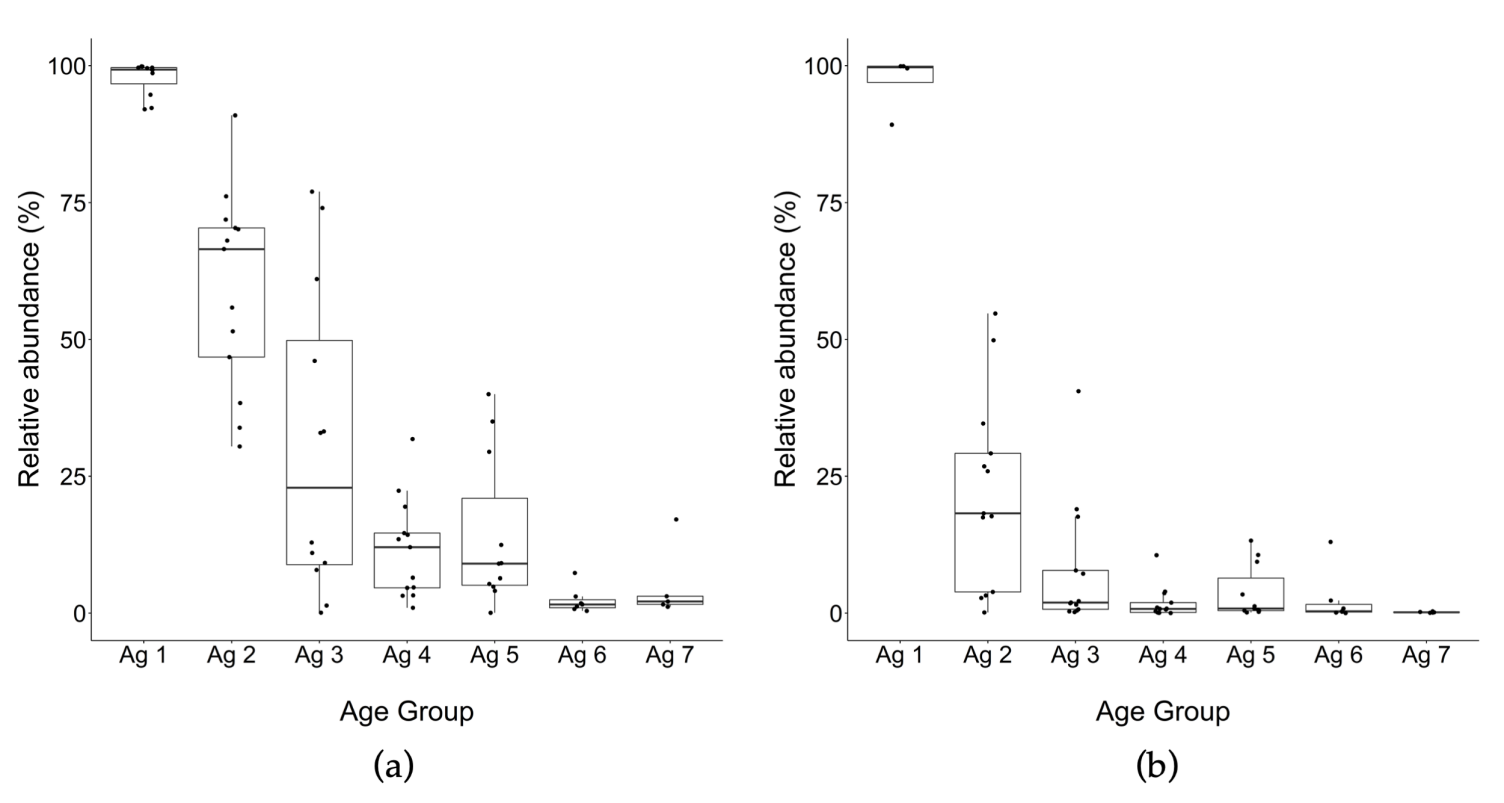
Box plot of relative abundance of *Lactococcus* spp. using OTU classification of 16S rRNA gene amplicon sequences for cheeses from trials 1 to 13 (% of total reads identified to genus level). (**a**) total DNA, *(***b**) PMA-treated DNA. Ag 1: 0–1 m, Ag 2: 3–6 m, Ag 3: 7–10 m, Ag 4: 12–15 m, Ag 5: 18–20 m, Ag 6: 24, Ag 7: 30–32 m. Each box ranges from Q1 (the first quartile) to Q3 (the third quartile) of the data distribution and the range represents the IQR (interquartile range). The median is indicated by a line across the box. The “whiskers” on box plots extend from Q1 and Q3 to the most extreme data points. In turn, each of the outliers is represented by a dot point.

The relative abundance of *Lactococcus* spp. was on average 97% of all the reads identified to genus level in the total DNA of all trials at 0–1 m (Figure 3 and Figure 4). This average abundance decreased to less than 60% at 3–6 m and to 26% at 7–10 m (Figure 3). The decline in abundance of *Lactococcus* spp. was slower after this point, gradually decreasing to 4% at 30–32 m. The average abundance for *Lactococcus* spp. from PMA-treated DNA was similar to total DNA (97%) but showed a sharper decline to 24% at 3–6 m, and to 7% at 7–10 m (Figure 3). The relative abundance of PMA-treated *Lactococcus* from 13–15 to 24 m remained between 2 and 4% average (with the exception of trial 5, which was at 16%) and at 30–32 m, reached almost zero. The relative abundance of *Lactococcus* spp. for PMA-treated DNA at 0–1 m was almost identical to total DNA, which suggests that at this point cell lysis/cell permeability of this species was not significant (Supplementary Material Table S3). Similar to total DNA, at 30–32 m, PMA-treated DNA from all five trials aged to this point was almost at zero. However, at intermediate ages (3–20 m), there was a significant difference between total DNA and PMA-treated DNA (Supplementary Material Table S4) and the difference between total DNA and PMA-treated DNA abundance reflecting the proportion of permeable cells ranged from a high of 35% at 3–6 m to less than 10% difference at later ripening ages (Figure 3), with a wide range among samples over 3–10 months (Figure 4, Supplementary Material Table S4). Overall, the average of *Lactococcus* spp. in 13 trials for both total and PMA-treated DNA significantly decreased from ages 0–1 to 3–6 m to 7–10, and to 13–15 m but after that the decreases were not significant. The difference between total and PMA-treated *Lactococcus* spp. DNA was significant in the middle of ripening (3–20 m) but at the beginning and the end point there was no significant difference between total and PMA-treated *Lactococcus* spp.

Total NSLAB abundance at 0–1 m ranged mostly from zero to a maximum of 8%, showing a steady increase up to 13–15 m of ripening (64–96%). In five out of seven trials reaching 24 m, NSLAB abundance attained 90–95%, followed by a decrease to 85–91% (Trial 3 had a 27% decrease, Supplementary Material Table S5). PMA-treated NSLAB had a sharp increase in relative abundance up to 7–10 m and reached 78–99% (except trial 3 where NSLAB were at 56%). From this point to 18–20 m, the increase slowed, reaching around 76–99%. From 18–20 m to 24 m, a decreasing trend was observed in three out of seven trials that were aged to this stage, reaching 80–97%. From 24 m to the end point, four out of five trials showed up to 14% decrease in NSLAB relative abundance, reaching 83–93% (Supplementary Material Table S5). The increase in NSLAB abundance was significant from the beginning to 13–15 m in total DNA and from the beginning to 7–10 m in PMA-treated DNA with no significant difference after that point. The difference between total and PMA-treated DNA was significant only in the intermediate age groups, 3–20 m, and not in other age groups (Supplementary Material Table S3).

**Figure 5:**
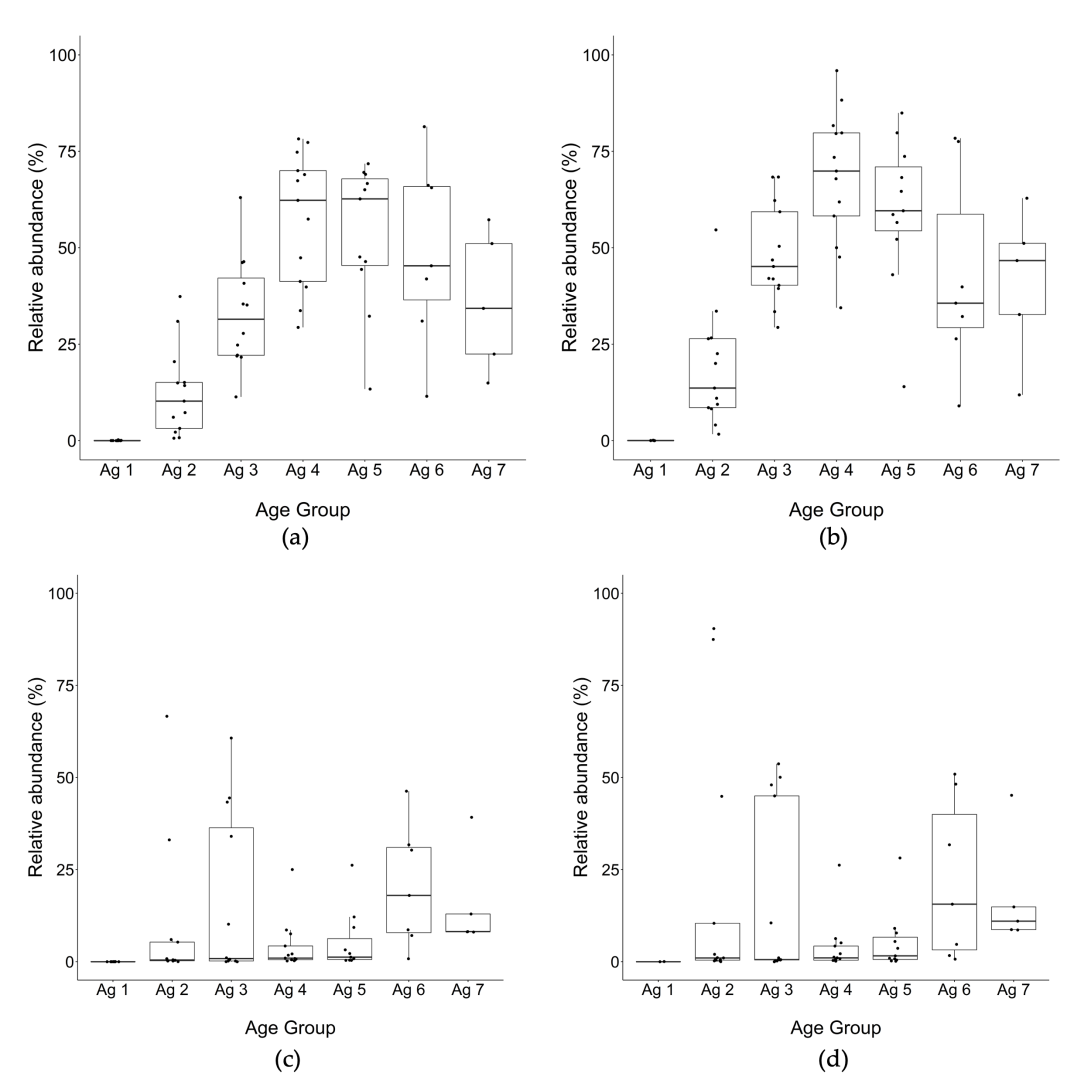
Relative abundance of (**a**,**b**) *Lacticaseibacillus,* and (**c**,**d**) *Lactobacillus* using OTU classification of 16S rRNA gene amplicon sequences for cheeses from trials 1 to 13 (% of total reads identified to genus level) (left): total DNA, (right): PMA-treated DNA. Ag 1: 0–1 m, Ag 2: 3–6 m, Ag 3: 7–10 m, Ag 4: 12–15 m, Ag 5: 18–20 m, Ag 6: 24, Ag 7: 30–32 m.

**Figure 6:**
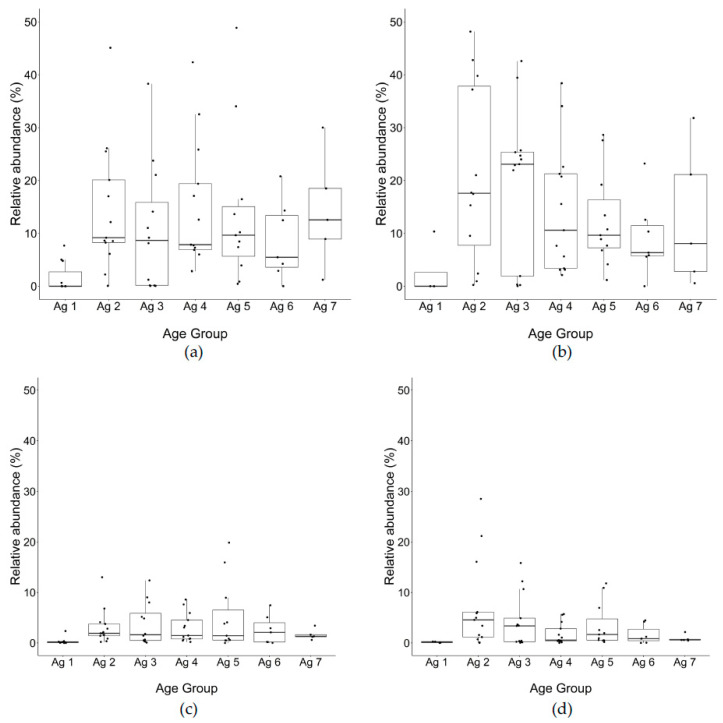
Relative abundance of (**a**,**b**) *Weissella* and (**c**,**d**) *Streptococcus* using OTU classification of 16S rRNA gene amplicon sequences for cheeses from 13 trials (% of total reads identified to genus level) (left): total DNA, (right): PMA-treated DNA. Ag 1: 0–1 m, Ag 2: 3–6 m, Ag 3: 7–10 m, Ag 4: 12–15 m, Ag 5: 18–20 m, Ag 6: 24, Ag 7: 30–32 m.

Among the NSLAB genera in this study, *Lacticaseibacillus* spp. was the most abundant genus (Figure 5a,b), attaining maximal relative abundance of 75% between 13 and 20 m, then the median abundance declined overall to just under 50% at 30–32 m. During the late ripening stages beyond 20 m, the relative abundance of *Lacticaseibacillus* spp. ranged widely from a minimum of 15% to a maximum of over 90% in separate trials. *Weissella* was the second most abundant NSLAB genus from the beginning of ripening to the end, and after 3–6 m remained at the same levels and showed little difference from total to PMA DNA (Figure 6a,b), suggesting they would be viable cells. *Streptococcus* was the next abundant taxon found in cheese at all time points (Figure 6c,d).

**Figure 7:**
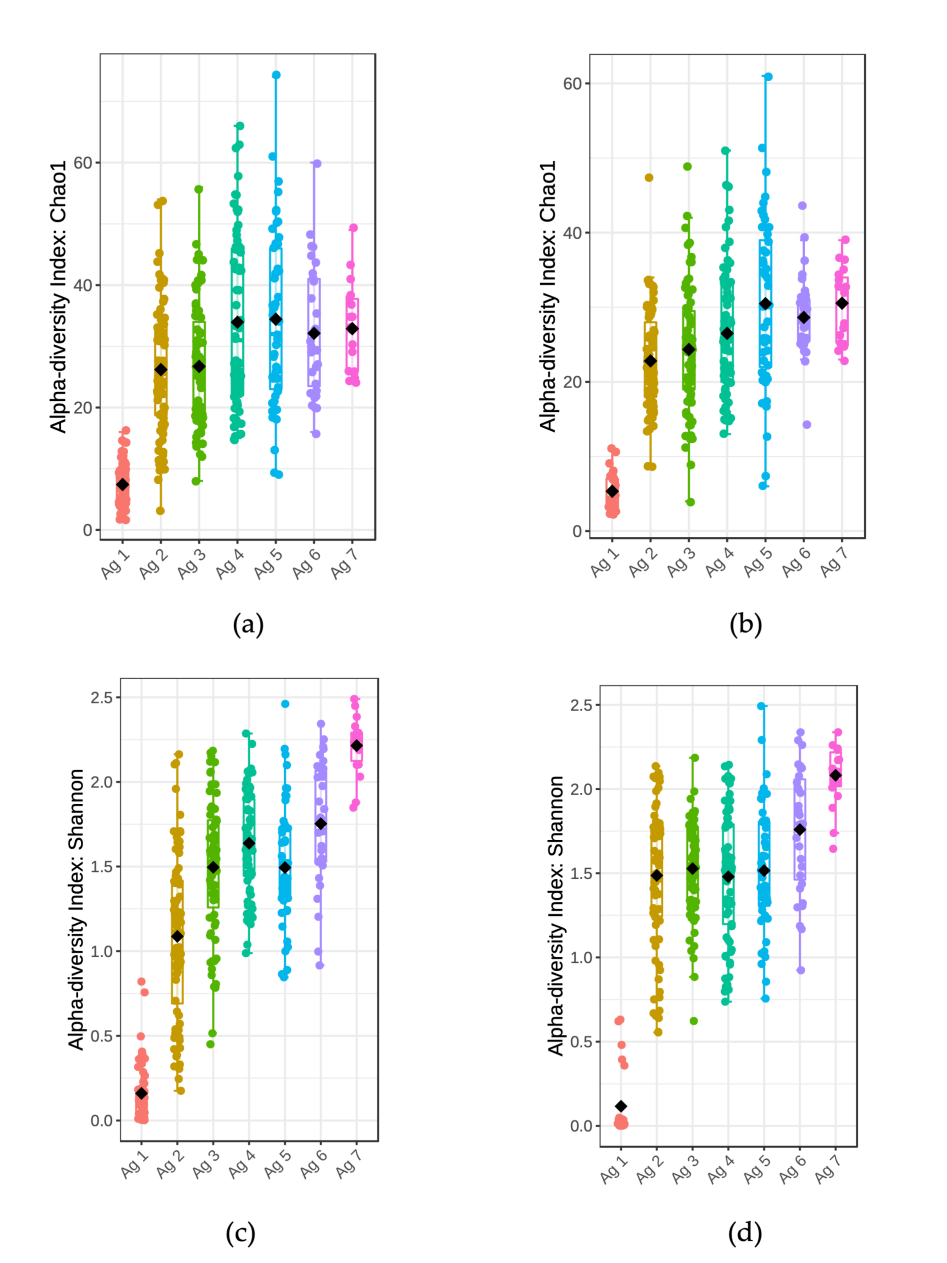
Measures of alpha diversity using 16S rRNA gene amplicon profiles at genus-level classification of OTUs generated from total and viable cell DNA (**a**,**c**): total DNA, (**b**,**d**): PMA-treated DNA) from 13 trials during 32 m of ripening. (**a**,**b**) Chao1 and (**c**,**d**) Shannon diversity index of each age group (indicated with a separate color). Ag 1: 0–1 m, Ag 2: 3–6 m, Ag 3: 7–10 m, Ag 4: 12–15 m, Ag 5: 18–20 m, Ag 6: 24 m, Ag 7: 30–32 m.

**Figure 8:**
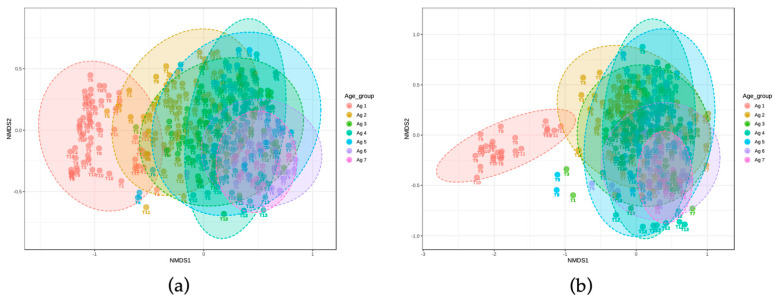
Beta diversity distances using the Bray Curtis measure based on OTU analysis according to age group and PMA treatment, (**a**) total DNA, (**b**) PMA-treated DNA. *p*-value indicates overall significant difference based on the Permanova test (*p* = 0.025).

For alpha diversity from the 16S rRNA gene amplicon relative abundance data, the Chao1 index was calculated for OTUs with evolutionary distance of 0.03 (or 97% 16S rRNA gene sequence similarity). The lowest diversity based on Chao1 index was observed at 0–1 m, where the majority of the population was identified to be *Lactococcus* spp. (Figure 7a,b). This diversity index increased over time up to 18–20 m and then attained a plateau from 24 m to 32 m. At every time point up to 24 m, the diversity was lower for PMA treated samples. The Shannon index represents sample diversity based on the number of species present (species richness) and the distribution of the number of organisms per species (species evenness). The median value of the Shannon index increased over time and was similar for PMA and non-PMA, indicating increasing richness and evenness of taxa (Figure 7c,d). Non-metric multidimensional scaling (NMDS) showed that the highest Bray–Curtis dissimilarity in beta diversity was found from age group 0–1 m in both PMA and non-PMA-treated samples (Figure 8). At later ages, there was a greater overlap between age groups indicating higher similarity in microbial population in older cheese samples compared to younger ones (except 0–1 m).

**Figure 9:**
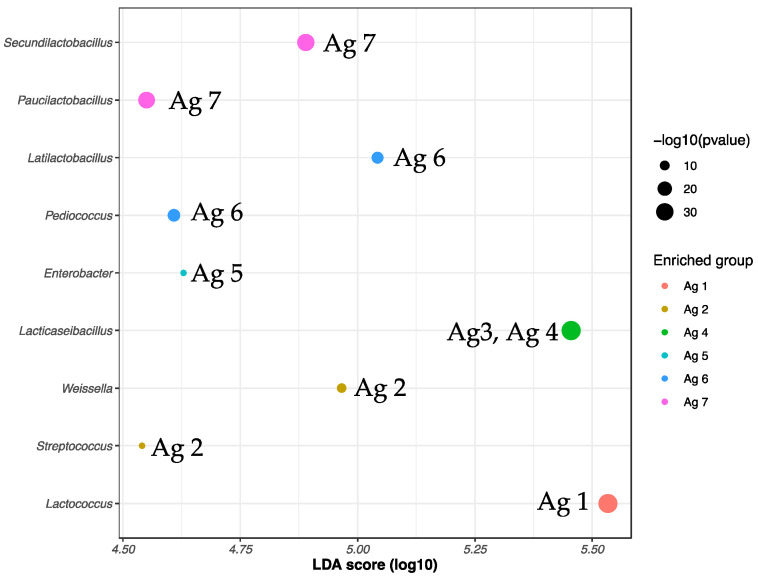
Linear discriminant analysis (LDA) of ASV abundance obtained from DNA extracted from PMA-treated bacterial cells of Cheddar cheese belonging to six out of seven ripening age categories sampled over 13 trials. Age 3 gave the same ASV biomarker as age 4 cheese microbiota.

From the ASV analysis carried out on 16S rRNA gene amplicon sequences, significant biomarker ASVs could be distinguished for six out of the seven age groups (Figure 9). Age 3 gave the same *Lacticaseibacillus* spp. biomarker ASVs as age 4 cheese microbiota. *Lactococcus* ASVs were significantly higher in abundance in age 1 cheeses (0–1 m), while four NSLAB genera were significant biomarkers for cheeses of ages 6 and 7 (*Pediococcus* and *Latilactobacillus* for age 6 while *Secundilactobacillus* and *Paucilactobacillus* were significantly enriched in age 7 cheeses). Over all trials, the number of ASVs per trial averaged 100 ± 14 for *Lactococcus* spp. (133 ASVs total) and 378 ± 65 ASVs per trial for *Lacticaseibacillus* spp. (520 ASVs total, Supplementary Material Tables S6–S8).

## 4. Discussion

Starter and non-starter lactic acid bacteria have distinct roles in modifying the physical and chemical characteristics of cheese during production (curd formation for SLAB) and ripening (both SLAB and NSLAB). The specific characteristics of the cheese depend on the microbial interactions in metabolizing proteins, fat, and residual carbohydrates [33]. By using both culture dependent methods and incorporating PMA treatment into the culture independent methods of 16S rRNA gene amplicon sequencing and PMA-qPCR, a comprehensive profile of Cheddar cheese microbiota and specific targets was obtained over a prolonged ripening period of 32 months.

Starters inoculated at ~6 log cfu/mL in cheese milk become concentrated to 7–9 log cfu/g of curd [34,35,36]. It is repeatedly reported that SLAB grow at the beginning of cheese manufacturing (cheese curd formation) and then gradually lose viability during ripening, mainly due to depletion of lactose, low moisture, low pH, non-optimum temperature, and high salt environment (pH 5.0–5.3, 5–13 °C and 4–6% salt in moisture) [37]. Their rate of decline depends on the processing steps (cooking temperature, salt rate, curd washing) that will affect the degree of autolysis according to the characteristics of the strains used in the starters [38]. Ganesan et al. [39] showed that *Lactococcus lactis* can remain metabolically active even though they are in a non-culturable state under carbohydrate starvation (i.e., after lactose is depleted at around 30 days of maturation in Cheddar).

Culturable counts of SLAB in cheese curd and at one month were 7–8 log cfu/g, around what has been reported. The sharpest decrease in SLAB viable counts was observed at 3–6 m (1–2 log decrease reaching 5.5–7 log cfu/g). The SLAB count at this age for most trials was similar to that found by Desfossés-Foucault et al. [36] and Crow et al. [40] who reported that culturable *Lactococcus* spp. counts decreased to 6–7 log cfu/g and 4–6 log cfu/g, respectively, after six months of ripening. However, the maximum decrease for this period in our study was 1–2 log, which was lower than the 2–3 log (from 9 to 6–7 log cfu/g) reported by Desfossés-Foucault et al. [36] and the 3–5-log decrease reported by Crow et al. [40]. Desfossés-Foucault et al. [41] reported a rapid decline in *L. lactis* subsp. *cremoris* from 9 to 7 log cfu/g of cheese after three days of accelerated ripening at 30 °C (in cheese slurry) and a total lack of detection on GM17 agar after six days (twelve days of simulated accelerated ripening was equivalent to six months at 4–6 °C). In our study, 10 starters were rotated through 13 trials where more similarity in rate of decline was found between starters used in the same trial than between trials for the same starter.

Through 16S rRNA gene amplicon sequencing, *Lactococcus* spp. comprised on average 97% of the community profile at the beginning of ripening (0–1 m)*,* due to the initial inoculation, growth in thermized milk, and concentration in the curd. Over time, the relative abundance of *Lactococcus* spp. decreased to 4% and near zero at 30–32 m for total and PMA-treated DNA, respectively. At 13–15 m and later, the changes in *Lactococcus* spp. from both total DNA and PMA-treated DNA were significantly less than the earlier time points. Compared to total DNA, PMA-treated DNA showed an earlier drastic decrease in *Lactococcus* (after 0–1 m), indicating that most cells detected at around 3–6 m were permeable. This drop in *Lactococcus* spp. abundance in PMA-treated DNA started earlier than was shown for the total DNA (3–6 m vs. 13–15 m), indicating that the permeabilized cells may accumulate at a higher rate than cell lysis (loss of total DNA).

Quantification by plate counts and qPCR using total DNA for *Lactococcus* spp. were consistent, showing the highest values at 0–1 m and decreasing thereafter. The difference between total and PMA-treated DNA was highest at 3–6 m (1.5–2.5 log), which means 150 to 300-fold ratio between intact to permeable *Lactococcus* spp. cells. SLAB plate count was the lowest, indicating the excluded DNA in PMA treatment was from the cells with damaged membranes at this point and were not able to grow on plates. This difference was lower after 3–6 m and remained the same at 1 log until 30–32 m. The low levels of difference between total and PMA-treated cells shows a low percentage of permeable *Lactococcus* spp. population with damaged membranes.

Comparing genome copy number and cDNA, Desfossés-Foucault et al. [36] showed a 100 to 1 ratio between dead/non-rRNA synthesizing to actively rRNA-synthesizing (cells at the beginning of ripening (m 0), which was interpreted as presence of higher number of dead but intact cells that do not synthesize RNA. The equal ratio between genome copy number and cDNA at one month of ripening was interpreted as lysis of those dead but intact cells [36,42]. However, based on the results reported in the above study, there is an increase in genome DNA from 2 to 5 m and the difference was again at around 100–fold for genome copy to cDNA, which is similar to the result of total and PMA at the same age in our study. The increase in genome copy number and higher ratio of genome DNA to cDNA however, might show that the smaller difference between genome and cDNA observed at one month in the above study was not necessarily due to cell lysis and the question remained whether the difference between genome and cDNA continued to diverge after 6 m, justifying the current study of cheese up to 32 m of age.

In several of the trials, starter plate counts increased by up to 1.5 log between 3–6 m and 7–10 m. A significant increase in the starter count after the initial decline has not been previously reported. However, a parallel increase was not observed when measuring the genome copy number of *Lactococcus* spp. by qPCR. This indicates a shift in the ability of *Lactococcus* to form colonies on plates between samples at 3–6 m and at 7–10 m, even though genome copy number remained stable. Bacterial populations with cells able to enter a dormant state can survive during stress such as nutrient depletion, which can be reversible. Kim et al. [43] showed that dormant cell populations consist of dead cells and persister cells where the non-lysed particles that do not resuscitate are the dead cells, and the dormant cells that resuscitate are the persister cells. Starvation induces the formation of persister cells, which tolerate stress factors such as antibiotics, heat, and acid. Using an antibiotic treatment, Tatenhove-Pel et al. [44] showed that *L. lactis* MG1363 can form a persister subpopulation. Persister cell types have been generated by more than one kind of stress; for example, oxidative and acid stress [43]. At the beginning of ripening when starters are submitted to a decrease in pH, carbon starvation, a decrease in water activity, and a temperature downshift [45] they may survive by entering a persister state in which they do not grow or reproduce but maintain a minimal metabolism. This reduces competition for scarce resources but the increase after 6 m could be due to dormant cells that have resuscitated when nutritional conditions change or when the stress is removed [43].

Based on the decreasing levels of SLAB in most studies at over 6–12 m of ripening, it was expected that no viable *Lactococcus* spp. or very low counts would be obtained after the long ripening period of 30–32 m. However, we showed that the SLAB viable counts in these cheeses did not drop significantly at 32 m of ripening, which extends the observations by Xue et al. [4]. After prolonged ripening (30–32 m), SLAB were culturable from the cheeses in our study up to 7 log, which was only on average half a log lower than the starting point. The final total genome copy number of *Lactococcus* spp. by qPCR was in line with the counts on plate at the end of ripening. PMA-qPCR revealed that a significant proportion of the *Lactococcus* spp. cells were permeable, as permeable cells were 1.5 log gene copy numbers/g less than total DNA from all cells. The PMA treatment method does not discriminate according to the extent of cell permeability, so cells with lower levels of permeability might still be capable of resuscitating on plates.

It has been accepted that starter viability becomes less important in later stages of ripening and the main role of starters at that stage is release of intracellular enzymes. However, viable bacteria on GM17 agar were obtained from samples as late as 32 m of ripening. Cells able to grow on media can represent (1) a subpopulation of the starter lactic acid bacteria that could grow throughout ripening, (2) persister cells that can resuscitate in cheese, as suggested by an increase in viable counts, (3) the portion of the population that could be resuscitated under optimal conditions of plating and incubation along with no competition from NSLAB as the GM17 is selective against most NSLAB.

Initial NSLAB concentrations in cheese made with high quality milk and sanitary conditions is 2–3 log cfu/g or less but can increase over time and reach 6–8 log after 6 m [8,9,34,46]. Our study supports these observations as NSLAB increased in relative abundance in all cheeses made from thermized milk at 7–10 m then stabilized in subsequent ripening periods. Surviving NSLAB are adapted to the conditions of acidity (pH 4.9–5.3), osmotic, oxidative, and high salt concentrations, low temperature, low moisture, and nutrient specificity [9]. These stress factors lead to reduced viability of SLAB and potential autolysis during ripening, releasing enzymes and other cell components that can promote the growth of NSLAB by providing proteolytic products such as proteins, peptides, and amino acids that can be used as substrates for NSLAB growth and flavor development [9]. Owing to these growth substances and NSLAB tolerance to the cheese environmental conditions, their population slowly increases about 4 to 6 log cfu/g during the first few months of ripening, which can have a significant impact on the ripening [8]. The decline in relative abundance of *Lactococcus* spp. was concurrent with the increase in the NSLAB abundance and at around 3–6 m, the dominance started to shift from *Lactococcus* spp. to NSLAB.

The comparison between *Lactococcus* spp. and *Lactobacillus* spp. genome copy number at 30–32 m measured by qPCR showed a 1–2 log difference that can be translated to up to a 100-fold lower abundance of *Lactococcus* spp. at the end of ripening. The low proportion of *Lactococcus* at this stage of ripening is corroborated by all three methods of quantification of the *Lactococcus* genus or SLAB. The rate of starter autolysis can determine the dominance of NSLAB in semi hard cheeses, especially in Cheddar, Emmental, and Gruyere [47,48]. The effect of starter autolysis on the growth of lactobacilli was noted by Desfossés-Foucault et al. [36] toward the end of 6 m of ripening when a decrease in genome copy number for *Lactococcus* coincided with an increase in genome copy number of lactobacilli, particularly in cheeses made from thermized milk. In the present study, at the beginning of ripening, NSLAB (mainly *Lactobacillus*, as well as *Streptococcus*, and *Weissella* genera) constituted an average of 2% of all DNA sequence reads. The increase in the relative abundance of viable NSLAB was concurrent with the decline in the *Lactococcus* spp. abundance. The shift in dominance from *Lactococcus* spp. to NSLAB proceeded at a variable rate depending on the trial.

The relative abundance of *Lactococcus* spp. from PMA-treated DNA was almost identical to total DNA at the 0–1 m age in the trials that were tested which suggested that at this point cell permeability of this species is not perceptible. At 3–6 m of ripening, the difference between the relative abundance of total and PMA-treated cells for both *Lactococcus* spp. and NSLAB was maximal (around 40%), but in opposite directions. While viable cells of *Lactococcus* were lower than total copy numbers, the relative abundance of viable cells of NSLAB was higher than that measured by total DNA, indicating that viable NSLAB made up a greater proportion of the viable microbial population and that other species were becoming non-viable at a higher rate. This difference gradually decreased to negligible after 24 m. A lower autolysis of starter strains means potential availability of enzyme activity remaining within the cells, which may continue to convert substrates through enzyme activity. This could indicate that at the beginning of ripening bacteria become permeable at a higher rate due to the stress conditions imposed by pH and salt, but as ripening progresses, the sensitive permeable cells do not remain intact, lysing to release DNA which is not recuperated from the cheese during the cellular DNA isolation process, leaving only the less permeable intact cells.

Slow autolysis of NSLAB and decrease in the density is reported in prolonged ripening, releasing enzymes throughout the ripening process [38,49]. If they have not been added as an adjunct culture, the origin of *Lacticaseibacillus* species is presumably from raw milk, and they are initially in minor proportion compared to the complex microbiota of the raw milk [50]. In most trials in our study, after attaining maximal abundance between 13 and 24 months, decreased relative abundance of *Lacticaseibacillus* was observed in our study after 24 m using quantification of DNA. However, the microbial community increased in evenness at the end of ripening, and NSLAB as a group of genera remained at over 90% of the relative abundance until 30–32 m. Originating from the production and processing environment, the initial diversity of NSLAB may be high early in Cheddar ripening, but overall abundance is low compared to starter (low evenness). Using the RAPD technique on isolates cultivated from Cheddar cheese, Fitzsimons et al. [51] showed that 8-week-old Irish Cheddar cheese contained *L. paracasei*, *L. plantarum*, and *L. curvatus* [52], whereas *L. paracasei* (96.4%) was dominant in 9 to 24-month-old cheese [51]. Among the NSLAB genera in our study, *Lacticaseibacillus* was the most abundant genus up to 20 m, but the subsequent rate of decline was variable among trials. *Lacticaseibacillus* spp. was a significant ASV biomarker for cheeses at age 3 and 4 (3 to 15 m) but four other genera (*Pediococcus, Latilactobacillus, Secundilactobacillus,* and *Paucilactobacillus*) differentiated the potentially viable (from PMA-treated DNA) microbiota of cheeses from 24 to 32 months. Some of these genera have been associated with cheese gas defects, such as the heterofermentative *Secundilactobacillus* and *Paucilactobacillus* [53]. *Latilactobacillus curvatus*, in particular, exhibits facultative heterofermentative metabolism, capable of forming slits in Cheddar cheese when residual sugars such as galactose are present. However, *Limosilactobacillus* was only found as a taxon of rare prevalence and abundance (under 0.01% in under 10% of samples). Species such as *Limosilactobacillus fermentum* have been directly linked to slit development in Cheddar cheese [4]. No presence of slit defect was observed in any of the trial cheeses in our study. *Pediococcus* is frequently found as part of the NSLAB of a number of cheeses and can be added as adjunct culture (ALAB) for formation of flavor and calcium lactate crystals in Cheddar cheese [54]. The variable occurrence of *Pediococcus* in the late-ripened Cheddar cheese in our study could be correlated with variability in grading at these later stages. The presence of multiple potential defect-causing genera during late ripening underscores the importance of developing tools to predict the optimal ripening time for marketing cheeses, considering the substantial investment and potential loss of product.

*Weissella* spp. was the second most abundant genus found at constant levels throughout ripening, with no significant difference between total DNA to PMA-treated DNA, indicating that the cells were intact and presumably viable. The *Weissella* species are obligate heterofermenting, catalase-negative organisms that produce D- or DL-isomers of lactic acid as the main end-product of fermentation [55] and are capable of producing exopolysaccharides. EPS may be technologically significant in certain sectors of the dairy fermentation industry for their rheological and textural effects on products. Some studied strains of *Weissella* may contribute to increasing the moisture retention of cheese without significantly affecting the cheese primary proteolysis, but altering the peptide profile of the cheese and increasing amino acids [56]. *Weissella* was not used as an adjunct culture in this study, so they most probably originated from raw milk.

*Streptococcus* was the next abundant taxon found in cheese at all time points, which would have come from milk or previous cheese manufacturing runs, as it was not used as part of the Cheddar starter culture. In a recent study, *Streptococcus* was the major (6–7%) sequence variance in facilities with different sources of milk [16]. *Streptococcus* may be added as part of the starter in Cheddar cheese production in some regions of the world [57]. In a standard manufacturing procedure employing a 38 °C cook temperature, even very low levels (0.007%) of *S*. *thermophilus* combined with normal levels of the mesophilic starter (1.7%) resulted in increased rates of acid production and the formation of significant amounts of galactose (13 mmol kg cheese) [16]. Martley and Michel [58] reported that a high proportion of mature Cheddar cheeses exhibiting a pinkish coloration at or just beneath the surface contained significant levels of galactose, which was not observed in this study. Pink discoloration has been related to *Thermus* contamination of the milk [3,59].

Overall, both viable plate count and qPCR quantification revealed the extended survival of SLAB/*Lactococcus* species in 32-m ripened cheese not shown previously. The increase in relative abundance of NSLAB varied significantly among trial cheeses, which might have repercussions on the ripening ability of cheeses depending on trial. During the period from 3–6 m to 18–20 m, significant numbers of permeable SLAB cells are present, while the proportion of permeable *Lacticaseibacillus* and *Lactobacillus* spp. remains low (high viability), indicating their respective potential contributions to converting substrates. The combination of techniques can shift the focus among targets, highlighting the relative abundance of specific species or groups of species according to their physiological state of viability. While qPCR employs specific primers (reducing competition among targets), the use of 16S rRNA gene primers might favor the abundant taxa (SLAB at the beginning and NSLAB at the later stages of ripening) at the expense of lower abundant taxa. Moreover, in the interpretation of such data, potential PCR bias of preferential amplification, which is due to differential efficacy of primers toward target taxa, must be taken into consideration [60].

## 5. Conclusions

In conclusion, although the total NSLAB population does not decrease during late ripening stages, the changes in composition of the NSLAB community in Cheddar cheese older than 24 m are distinguished by a variable decrease in the abundance of *Lacticaseibacillus* spp. and the occurrence of potential defect-causing bacterial genera. Even though there has been significant progress toward detecting, quantifying, and understanding the status of the bacterial cells in cheese (e.g., permeabilization and autolysis of cells), a holistic population-based approach is needed in order to elucidate the interactions between various subpopulations in the cheese during ripening. This view should consider all cell types, from culturable cells to persister cells and lysed cells as well as the interaction of the cheese community in terms of functional metabolism.

The 16S rRNA gene amplicon sequencing poses limits to the resolution of intra-species sequence diversity. A more thorough description of genotypic variation within species using shotgun metagenomics [61] will help delineate the metabolic potential and diversity within species during long periods of ripening. Moreover, shotgun metagenomics will enable better characterization of microbial communities within cheese [18,62], inferring functional information [63], enriching taxonomic profiling with an unprecedented depth up to species and strain level variation that could affect the flavor of cheese, and discovering phage abundance [64]. However, a primary challenge facing this approach is the difficulty in assembling genomes from highly diverse sequences of cheese microbiota using as reference the available genomes deposited in widely used public databases [65].

## Data Availability

Not applicable.

## Supplementary Materials

The following supporting information can be downloaded at: https://www.mdpi.com/article/10.3390/microorganisms10081669/s1. Figure S1. A total of 13 cheese making trials were conducted over the course of 32 m. Figure S2. Average viable counts (log cfu/g cheese) of SLAB plated on GM17 agar for the two starters used in each of 13 trials over a maximum of 32 months of ripening. Figure S3. Average counts (log cfu/g cheese) on (a) GM17 agar to select for SLAB and (b) MRSv agar to select for NSLAB for 13 trials over a maximum of 32 m of ripening. Table S1. Average viable counts of microbial groups plated on GM17 agar to select for SLAB and MRSv for NSLAB over a maximum of 32 months of ripening for 13 trials. Table S2. Average 16S rRNA log gene copy number of specific target bacterial genera per g of cheese from total and viable cells in cheese samples from five trials measured by qPCR during 32 months of ripening. Table S3. Average relative abundance of *Lactococcus* spp. and NSLAB using 16S rRNA gene amplicon sequencing (% of total reads identified to genus level). Table S4. Average relative abundance of *Lactococcus* spp. using 16S rRNA gene amplicon sequencing per trial (% of total reads identified to genus level) over a maximum of 32 months of ripening. Table S5. Average relative abundance of NSLAB using 16S rRNA gene amplicon sequencing per trial (% of total reads identified to genus level per trial) over a maximum of 32 months of ripening. Table S6. Number of ASVs. Table S7. Total number of ASVs for *Lactococcus* spp. and *Lacticaseibacillus* spp. across 13 trials. Table S8. Number of ASVs for *Lactococcus* spp. and *Lacticaseibacillus* spp. for each of 13 trials.
