# Supplementary material for: Dynamics of Starter and Non-Starter Lactic Acid Bacteria Populations in Long-Ripened Cheddar Cheese Using Propidium Monoazide (PMA) Treatment
Source: Microorganisms. 2022 Aug 19;10(8):1669. doi: 10.3390/microorganisms10081669 (PMC9413250; doi:10.3390/microorganisms10081669)
Supplement: Supplementary file 1 [file microorganisms-10-01669-s001.zip › microorganisms-1828874-supplementary.pdf]

microorganisms

MDPI

Supplementary Material for Barzideh et al. (2022). Long-ripened Cheddar cheese microbiota.

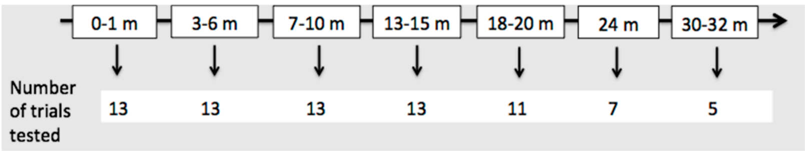

		Age (month)							
**Experiment**	**Number of trials/vats**	**0-1**	**3-6**	**7-10**	**13-15**	**18-20**	**24**	**30-32**	**Total**
Plate count	Number of trials	13	13	13	13	11	7	5	13
	Number of vats	74	72	71	69	51	30	18	385
MiSeq 16S amplicon sequencing	Number of trials	11 (total) 4 (PMA)	13	13	13	11	7	5	13
	Number of vats (Non PMA)	62	76	71	69	49	31	18	376
	Number of vats (PMA)	24	75	71	69	49	31	18	337
	Total	86	151	142	138	98	62	36	713
qPCR	Number of trials	1-5	1-5	1-5	1-5	1-5	1-5	1-5	5
	Number of vats (both total and PMA)	52	60	52	50	38	38	36	326

Figure S1. A total of 13 cheese making trials were conducted over the course of 32 m. Cheeses from all 13 trials were sampled at 0-1 m, 3-6 m, 7-10 m, and at 13-15 m while cheeses from 11 trials were sampled at 18-20 m, from 7 trials at 24 m and from 5 trials at 30-32 m time points. Cheeses from all trials were subjected to viable plate count, while MiSeq data were obtained on total DNA for 11 trials and PMA treatment was carried out on cheeses from trials 8-11). For qPCR, cheeses from trials 1-5 were analyzed with and without PMA treatment for all time points. No cheeses with slit defect were found during the course of this study.

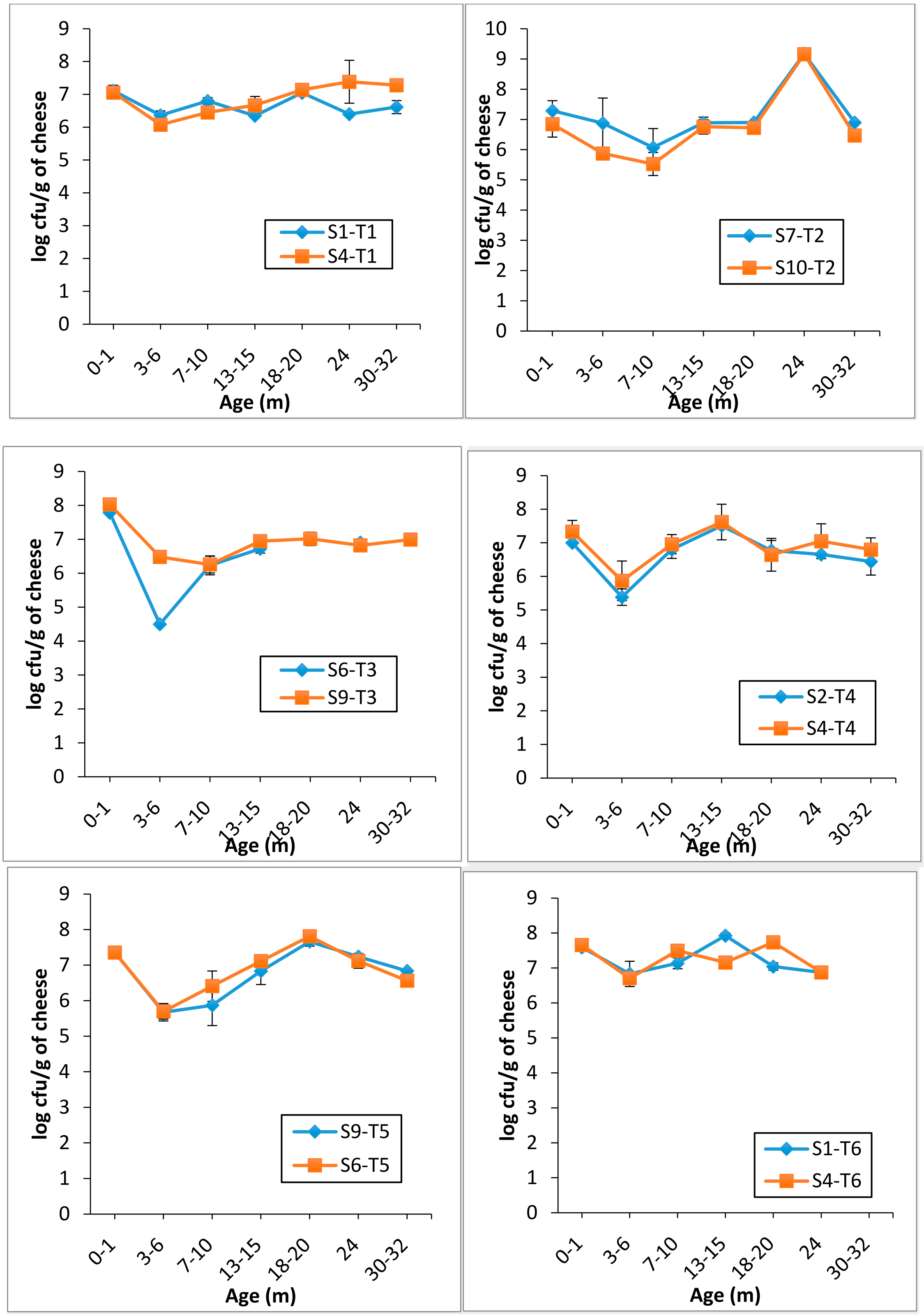
log cfu/g of cheese
Age (m)
0-1
3-6
7-10
13-15
18-20
24
30-32
S1-T1
S4-T1
S7-T2
S10-T2
S6-T3
S9-T3
S2-T4
S4-T4
S9-T5
S6-T5
S1-T6
S4-T6

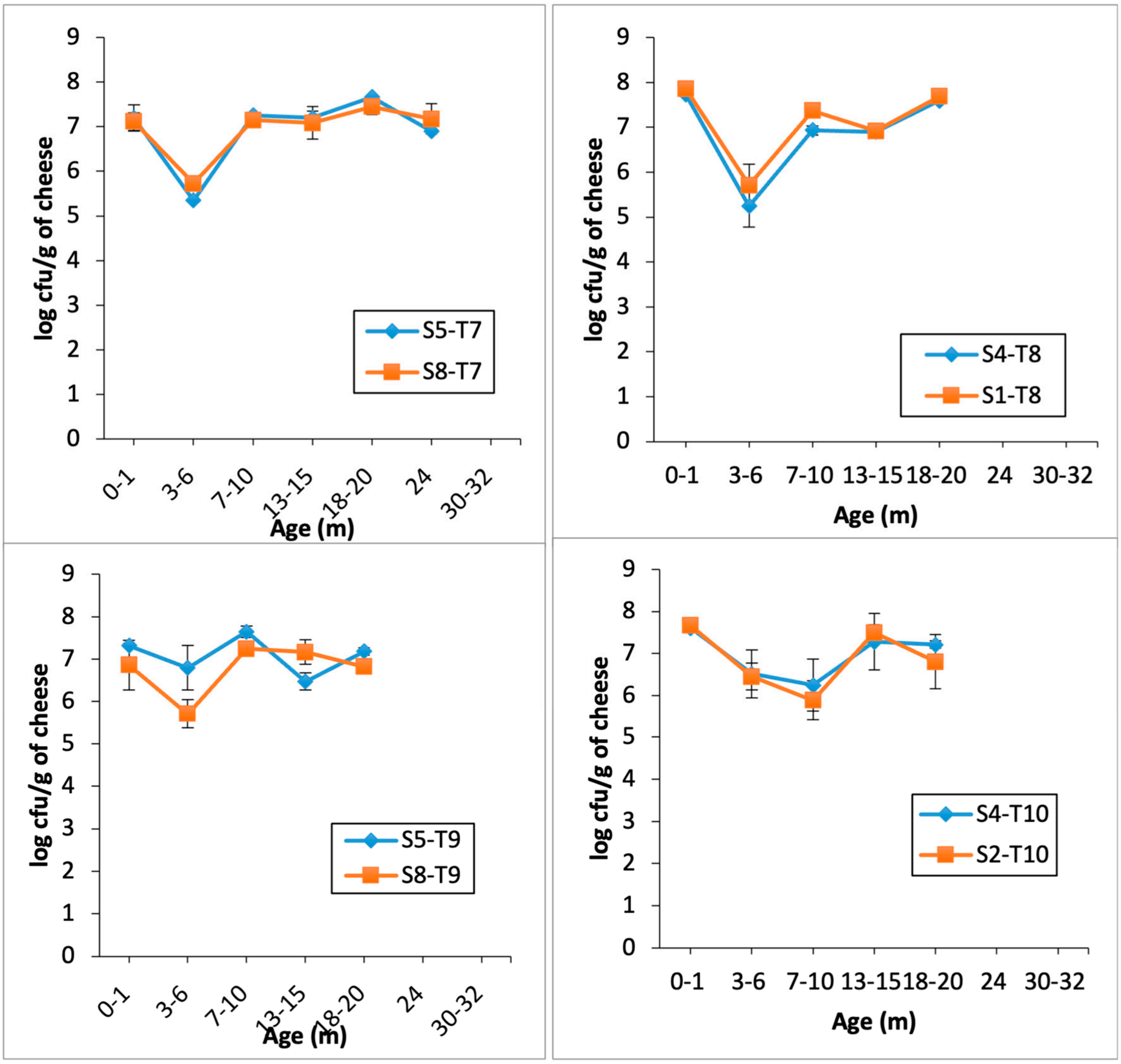
log cfu/g of cheese
Age (m)
0-1
3-6
7-10
13-15
18-20
24
30-32
S5-T7
S8-T7
S4-T8
S1-T8
S5-T9
S8-T9
S4-T10
S2-T10

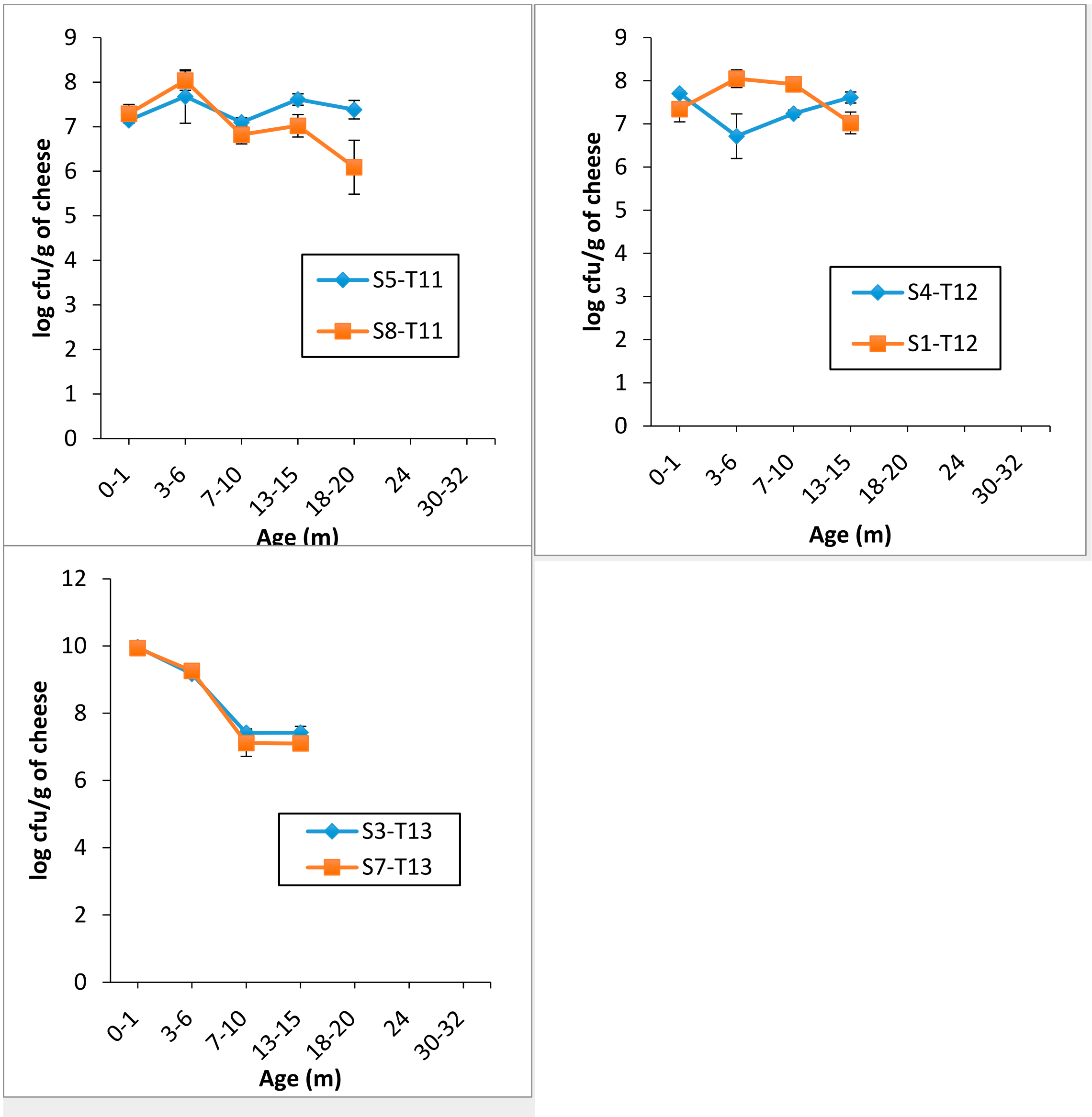

Figure S2. Average viable counts (log cfu/g cheese) of SLAB plated on GM17 agar for the two starters used in each of 13 trials over a maximum of 32 months of ripening. Sampling was performed over a course of 32 m at 0-1 m (13 trials), 3-6 m (13 trials), 7-10 m (13 trials), 13-15 m (13 trials), 18-20 m (11 trials), 24 m (7 trials) and 30-32 m (5 trials) time points. In trial 3, two data points are missing for starter S6 at 18-20 m and 32 m, because these cheeses were not provided by the company.

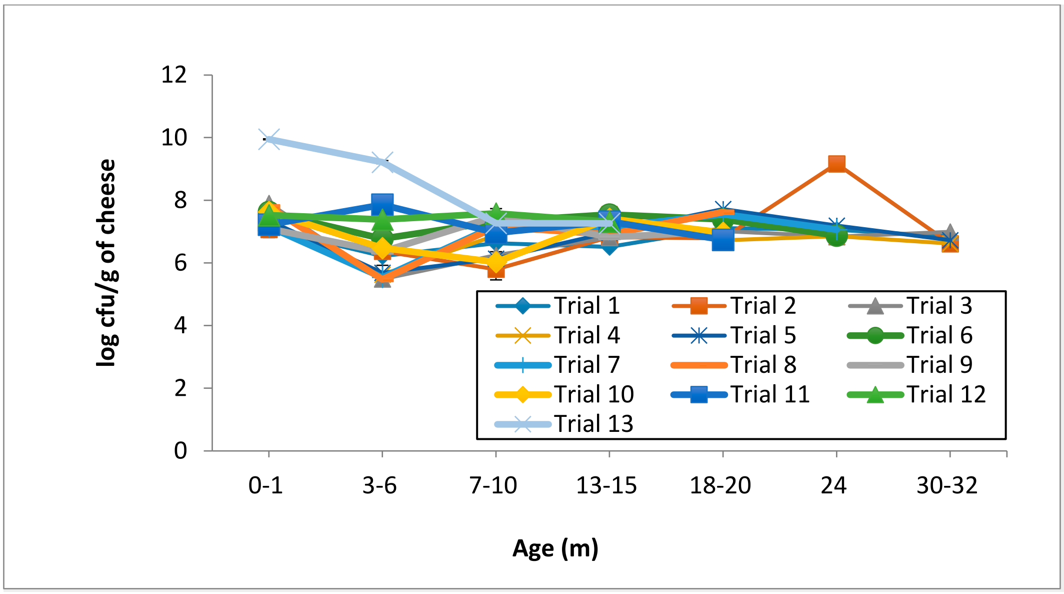

(a)

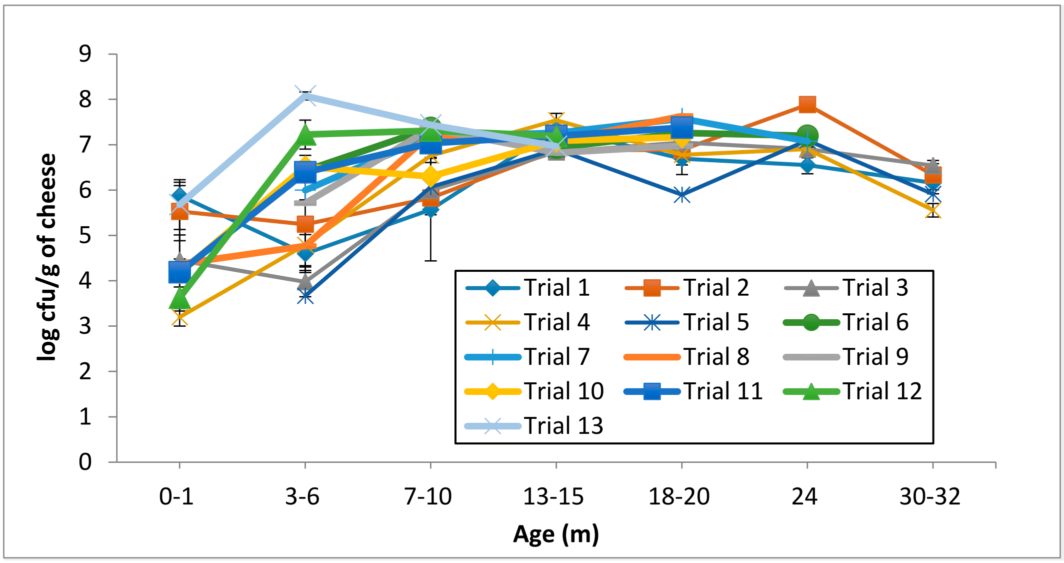

(b)

Figure S3. Average counts (log cfu/g cheese) on (a) GM17 agar to select for SLAB and (b) MRSv agar to select for NSLAB for 13 trials over a maximum of 32 m of ripening. Standard error applied to each data point. Sampling was performed over a course of 32 m at 0-1 m (13 trials), 3-6 m (13 trials), 7-10 m (13 trials), 13-15 m (13 trials), 18-20 m (11 trials), 24 m (7 trials) and 30-32 m (5 trials) time points.

Table S1. Average viable counts of microbial groups plated on GM17 agar to select for SLAB and MRSv for NSLAB over a maximum of 32 months of ripening for 13 trials a. average of all trials b. SLAB individual trials, and c. NSLAB individual trials.

a.

	Age (months)						
**Group of taxa**	**0-1**	**3-6**	**7-10**	**13-15**	**18-20**	**24**	**30-32**
SLAB counts on GM17	7.58 ± 0.21^{a*}	6.50 ± 0.31^{b}	6.82 ± 0.16b^{c}	7.11 ± 0.09^{c}	7.15 ± 0.11^{c}	7.29 ± 0.32^{acd}	6.76 ± 0.07^{bd}
NSLAB counts on MRSv	3.16 ± 0.64^{a}	5.65 ± 0.36^{b}	6.73 ± 0.19^{bc}	7.08 ± 0.06^{c}	7.03 ± 0.15^{c}	7.09 ± 0.15^{ad}	6.10 ± 0.17^{bd}

b.

	Age (months)						
**Trial number**	**0-1**	**3-6**	**7-10**	**13-15**	**18-20**	**24**	**30-32**
1	7.08 ± 0.09$^{a, A}$	6.22 ± 0.10$^{b, AB}$	6.63 ± 0.13^{ab,ABCDE}	6.51 ± 0.15^{ab,A}	7.11 ± 0.07$^{ab, A}$	7.06 ± 0.50$^{ab, A}$	6.84 ± 0.25$^{ab, A}$
2	7.07 ± 0.26$^{a, A}$	6.37 ± 0.45$^{a, AB}$	5.80 ± 0.34$^{a, A}$	6.82 ± 0.13$^{a, AB}$	6.78 ± 0.11$^{a, A}$	9.16 ± 0.03$^{b, B}$	6.61 ± 0.16$^{a, A}$
3	7.90 ± 0.06$^{a, C}$	5.49 ± 0.45$^{bc, A}$	6.25 ± 0.17$^{bc, ABCD}$	6.81 ± 0.1$^{bc, AB}$	7.01 ± 0.20$^{ac, A}$	6.84 ± 0.02$^{bc, ,A}$	6.99 ± 0.03$^{c, A}$
4	7.17 ± 0.17$^{a, A}$	5.63 ± 0.30$^{b, A}$	6.88 ± 0.18$^{b, BCDEF}$	7.56 ± 0.24$^{b, B}$	6.72 ± 0.23$^{b, A}$	6.85 ± 0.24$^{b, A}$	6.62 ± 0.24$^{ab, A}$
5	7.36 ± 0.04$^{a, ABC}$	5.69 ± 0.15$^{a, A}$	6.14 ± 0.34$^{bc, ABC}$	6.97 ± 0.18$^{c, AB}$	7.72 ± 0.08$^{b, A}$	7.19 ± 0.08$^{c, A}$	6.72 ± 0.09$^{b, A}$
6	7.63 ± 0.06$^{a, ABC}$	6.77 ± 0.17$^{b, ABC}$	7.32 ± 0.12$^{ab, EF}$	7.54 ± 0.19$^{ab, B}$	7.39 ± 0.16^{ab,A}	6.88 ± 0.02$^{c, A}$	-
7	7.15 ± 0.16$^{a, A}$	5.54 ± 0.10$^{b, A}$	7.19 ± 0.04$^{a, EF}$	7.14 ± 0.18$^{a, AB}$	7.56 ± 0.09$^{a, A}$	7.06 ± 0.20$^{a, A}$	-
8	7.80 ± 0.08$^{a, BC}$	5.48 ± 0.33$^{b, A}$	7.15 ± 0.12$^{cd, DEF}$	6.91 ± 0.02$^{c, AB}$	7.62 ± 0.07$^{ad, A}$	-	-
9	7.09 ± 0.28$^{ab, A}$	6.36 ± 0.40$^{a, AB}$	7.49 ± 0.13$^{b, EF}$	6.82 ± 0.22$^{ab, AB}$	7.00 ± 0.09$^{ab, A}$	-	-
10	7.63 ± 0.05$^{a, ABC}$	6.48 ± 0.25$^{bc, AB}$	6.03 ± 0.33$^{c, AB}$	7.41 ± 0.24$^{ab, B}$	6.96 ± 0.36$^{abc, A}$	-	-
11	7.22 ± 0.1$^{a, AB}$	7.86 ± 0.30$^{a, C}$	6.96 ± 0.12$^{a, CDEF}$	7.32 ± 0.18$^{a, AB}$	6.73 ± 0.40$^{a, A}$	-	-
12	7.53 ± 0.16$^{a, ABC}$	7.38 ± 0.39$^{a, C}$	7.58 ± 0.16$^{a, F}$	7.32 ± 0.18$^{a, AB}$	-	-	-
13	9.95 ± 0.01$^{a, C}$	9.22 ± 0.05$^{b, D}$	7.27 ± 0.20$^{c, EF}$	7.26 ± 0.14$^{c, AB}$	-	-	-

c.

	Age (months)						
**Trial number**	**0-1**	**3-6**	**7-10**	**13-15**	**18-20**	**24**	**30-32**
1	5.88 ± 0.22$^{a, A}$	4.60 ± 0.42$^{a, AB}$	5.57 ± 1.13$^{ab, A}$	7.35 ± 0.06$^{b, AB}$	6.69 ± 0.35$^{ab, B}$	6.55 ± 0.19$^{ab, A}$	6.16 ± 0.24$^{ab, AB}$
2	5.53 ± 0.65$^{ab, AB}$	5.25 ± 0.53$^{ab, ABC}$	5.83 ± 0.37$^{ab, AB}$	6.87 ± 0.14$^{a, AB}$	6.88 ± 0.09$^{a, BC}$	7.88 ± 0.02$^{b, C}$	6.33 ± 0.32$^{ab, B}$
3	4.45 ± 0.96$^{a, DE}$	3.98 ± 0.33$^{a, A}$	5.99 ± 0.23$^{bc, AB}$	6.84 ± 0.13$^{bc, A}$	7.05 ± 0.17$^{bc, BCD}$	6.90 ± 0.05$^{b, AB}$	6.54 ± 0.04$^{c, B}$
4	3.20 ± 0.2$^{a, CD}$	4.79 ± 0.46$^{d, ABC}$	6.76 ± 0.15$^{b, AB}$	7.54 ± 0.15$^{c, B}$	6.78 ± 0.22$^{bc, B}$	6.90 ± 0.19$^{bc, AB}$	5.56 ± 0.15$^{d, A}$
5	BDL	3.67 ± 0.37$^{a, A}$	6.07 ± 0.29$^{bc, AB}$	6.90 ± 0.16$^{b, AB}$	5.90 ± 0.14$^{c, A}$	7.10 ± 0.09$^{b, AB}$	5.91 ± 0.17$^{c, AB}$
6	BDL	6.43 ± 0.17$^{a, CD}$	7.37 ± 0.06$^{b, B}$	6.93 ± 0.09$^{a, AB}$	7.26 ± 0.07$^{b, BCD}$	7.20 ± 0.19$^{b, B}$	-
7	BDL	6.00 ± 0.21$^{a, BCD}$	7.18 ± 0.04$^{b, AB}$	7.26 ± 0.07^{b,AB}	7.58 ± 0.08$^{c, CD}$	7.08 ± 0.18$^{bc, AB}$	-
8	4.37 ± 0.11$^{a, ABC}$	4.77 ± 0.54$^{b, A}$	7.23 ± 0.12$^{bc, B}$	7.07 ± 0.06$^{c, AB}$	7.63 ± 0.04$^{b, D}$	-	-
9	BDL	5.71 ± 0.34$^{a, BCD}$	7.36 ± 0.05$^{b, B}$	6.82 ± 0.22$^{ab, A}$	6.98 ± 0.07$^{a, BCD}$	-	-
10	4.17 ± 0.84$^{a, ABC}$	6.52 ± 0.25$^{b, CDE}$	6.30 ± 0.42$^{ab, AB}$	7.08 ± 0.20$^{b, AB}$	7.17 ± 0.23$^{b, BCD}$	-	-
11	4.19 ± 0.15$^{a, ABC}$	6.39 ± 0.12$^{b, CD}$	7.04 ± 0.09$^{c, AB}$	7.19 ± 0.14$^{c, AB}$	7.38 ± 0.08$^{c, BCD}$	-	-
12	3.63 ± 0.24$^{a, BCD}$	7.23 ± 0.32$^{b, DE}$	7.31 ± 0.13$^{b, B}$	7.19 ± 0.14$^{b, AB}$	-	-	-
13	5.68 ± 0.55$^{a, AB}$	8.08 ± 0.09$^{b, E}$	7.44 ± 0.10^{a,B}	6.97 ± 0.14$^{a, AB}$	-	-	-

* Different lower case letters (a-d) indicate significant differences between age groups within the same trial ($p \leqq 0.05$) and different capital letters (A-F) indicate significant differences between trials within the same age group ($p \leqq 0.05$) in sections b and c. The statistical comparisons were carried out using Tukey's Honestly Significant Difference test (where there were no violations of the test assumptions for the homogeneity of variance, Levene's k - sample comparison of variances or 1 - way ANOVA using the Games - Howell post-hoc analysis where the test assumptions for the homogeneity of variance were violated). Data are the mean values ± standard error from at least three separate samples. BDL: below detection level; no isolates obtained on -1 dilution plates.

Table S2. Average 16S rRNA log gene copy number of specific target bacterial genera per g of cheese from total and viable cells in cheese samples from five trials measured by qPCR during 32 months of ripening. Standard error applied to each data point. a. *Lactococcus* spp., *Lactobacillus* spp.; and *Lacticaseibacillus* spp. average of all trials, b. *Lactococcus* spp. individual trials, c. *Lactobacillus* spp. individual trials; and d. *Lacticaseibacillus* spp. individual trials.

a.

	Age (months)						
**Group of taxa**	**0-1**	**3-6**	**7-10**	**13-15**	**18-20**	**24**	**30-32**
Lactococcus spp. Total	9.03 ± 0.08^{a}	7.51 ± 0.13$^{b, A}$	6.16 ± 0.28$^{cd, A}$	5.26 ± 0.24$^{cd, A}$	5.38 ± 0.2$^{cd, A}$	6.05 ± 0.12$^{c, A}$	5.23 ± 0.18$^{d, A}$
Lactococcus spp. Viable	-	5.43 ± 0.13$^{a, B}$	5.40 ± 0.19$^{a, B}$	4.43 ± 0.13$^{b, B}$	4.40 ± 0.11$^{b, B}$	4.69 ± 0.11$^{b, B}$	4.10 ± 0.16$^{b, B}$
Lactobacillus spp. Total	4.39 ± 0.19^{a}	5.93 ± 0.16$^{b, A}$	6.13 ± 0.2$^{8b, A}$	6.15 ± 0.18$^{b, A}$	6.58 ± 0.17$^{cd, A}$	7.12 ± 0.15$^{d, A}$	6.59 ± 0.17$^{cd, A}$
Lactobacillus spp. Viable	-	4.80 ± 0.2$^{a, B}$	6.61 ± 0.16$^{b, A}$	5.92 ± 0.13$^{c, A}$	6.33 ± 0.19$^{bc, A}$	6.95 ± 0.1$^{b, A}$	6.24 ± 0.11$^{bc, A}$
Lacticaseibacillus spp. Total	3.70 ± 0.12^{a}	4.74 ± 0.32$^{abc, A}$	5.70 ± 0.27$^{b, A}$	4.14 ± 0.26$^{ac, A}$	5.65 ± 0.37$^{b, A}$	5.46 ± 0.1$^{bc, A}$	4.59 ± 0.21$^{abc, A}$
Lacticaseibacillus spp. Viable	-	4.08 ± 0.34$^{ab, A}$	5.28 ± 0.45$^{ab, A}$	4.32 ± 0.16$^{b, A}$	5.42 ± 0.4a$^{b, A}$	5.21 ± 0.13$^{b, A}$	4.23 ± 0.19$^{a, A}$

b.

	Age (months)						
**Trial number**	**0-1**	**3-6**	**7-10**	**13-15**	**18-20**	**24**	**30-32**
1	8.33 ± 0.12$^{a, A}$	7.16 ± 0.25$^{b, AB}$	6.40 ± 0.34$^{bc, AB}$	5.56 ± 0.22$^{cd, AB}$	5.66 ± 0.05$^{c, AB}$	6.33 ± 0.04$^{bd, AB}$	5.33 ± 0.47$^{abcd, A}$
2	8.82 ± 0.17$^{a, B}$	6.60 ± 0.22$^{b, A}$	6.36 ± 0.25$^{bc, A}$	5.15 ± 0.05$^{de, AB}$	5.46 ± 0.12$^{cd, AB}$	6.64 ± 0.2$^{b, A}$	4.48 ± 0.13$^{e, A}$
3	9.10 ± 0.06$^{a, BC}$	7.62 ± 0.16$^{b, BC}$	5.67 ± 0.98$^{abc, AB}$	6.8 ± 0.18$^{bc, A}$	5.06 ± 1.06$^{abc, AB}$	5.93 ± 0.24$^{c, AB}$	6.53 ± 0.04$^{c, B}$
4	9.40 ± 0.09$^{a, C}$	7.98 ± 0.11$^{a, C}$	5.17 ± 0.19$^{bc, B}$	4.62 ± 0.6$^{c, B}$	4.75 ± 0.24$^{c, A}$	6.18 ± 0.23$^{b, AB}$	5.09 ± 0.33$^{bc, A}$
5	9.19 ± 0.05$^{a, BC}$	8.16 ± 0.1$^{b, C}$	7.36 ± 0.15$^{c, A}$	4.49 ± 0.33$^{d, B}$	6 .00 ± 0.21$^{e, B}$	5.53 ± 0.12$^{de, B}$	4.94 ± 0.12$^{d, A}$
1 - PMA	-	5.54 ± 0.16$^{a, A}$	5.61 ± 0.13$^{a, A}$	4.56 ± 0.29$^{a, AB}$	5.24 ± 0.04$^{a, A}$	5.33 ± 0.1$^{a, A}$	4.61 ± 0.55$^{a, A}$
2 - PMA	-	5.04 ± 0.18$^{a, A}$	4.64 ± 0.41$^{a, A}$	4.61 ± 0.09$^{a, B}$	4.15 ± 0.19$^{a, AB}$	5.20 ± 0.12$^{a, A}$	4.13 ± 0.19$^{a, A}$
3 - PMA	-	5.23 ± 0.55$^{a, A}$	5.68 ± 0.65$^{a, A}$	4.43 ± 0.37$^{a, AB}$	4.46 ± 0.07$^{a, B}$	4.72 ± 0.26$^{a, AB}$	4.06 ± 0.05$^{a, A}$
4 - PMA	-	5.53 ± 0.12$^{a, A}$	4.88 ± 0.13$^{b, A}$	4.65 ± 0.35$^{abc, AB}$	4.34 ± 0.19$^{b, B}$	4.24 ± 0.06$^{c, B}$	4.48 ± 0.49$^{abc, A}$
5 - PMA	-	5.80 ± 0.13$^{a, A}$	6.02 ± 0.22$^{a, A}$	4.01 ± 0.14$^{bc, B}$	4.05 ± 0.16$^{bc, B}$	4.33 ± 0.04$^{b, B}$	3.61 ± 0.2$^{c, A}$

c.

	Age (months)						
**Trial number**	**0-1**	**3-6**	**7-10**	**13-15**	**18-20**	**24**	**30-32**
1	5.35 ± 0.43$^{a, AB}$	6.23 ± 0.28$^{ab, A}$	6.90 ± 0.20$^{b, A}$	7.10 ± 0.15$^{b, A}$	6.47 ± 0.09$^{ab, BC}$	7.53 ± 0.07$^{b, A}$	6.87 ± 0.37$^{b, AB}$
2	5.50 ± 0.65$^{a, AB}$	5.58 ± 0.47$^{a, A}$	6.08 ± 0.38$^{a, A}$	6.33 ± 0.09$^{a, B}$	6.43 ± 0.12$^{a, BC}$	6.30 ± 0.5$^{a, AB}$	5.70 ± 0.15$^{a, A}$
3	4.48 ± 0.07$^{a, A}$	6.23 ± 0.37$^{bc, A}$	5.25 ± 1.05$^{abc, A}$	6.30 ± 0.18$^{b, AB}$	7.60 ± 0.21$^{c, A}$	7.70 ± 0.15$^{c, A}$	7.70 ± 0.12$^{c, B}$
4	3.43 ± 0.13$^{a, B}$	6.42 ± 0.19$^{bc, A}$	6.05 ± 0.16$^{b, A}$	6.60 ± 0.13$^{bc, AB}$	7.02 ± 0.21$^{c, AB}$	7.18 ± 0.29$^{c, AC}$	6.40 ± 0.23$^{bc, A}$
5	3.87 ± 0.07$^{a, B}$	5.18 ± 0.25$^{b, A}$	6.60 ± 0.45$^{bc, A}$	4.83 ± 0.25$^{ab, C}$	5.68 ± 0.18$^{bc, C}$	6.84 ± 0.15$^{c, BC}$	6.44 ± 0.17$^{c, A}$
1 - PMA	-	5.05 ± 0.33$^{a, AB}$	6.88 ± 0.34$^{b, A}$	6.48 ± 0.38$^{ab, A}$	6.70 ± 0.06$^{b, A}$	7.17 ± 0.09$^{b, AB}$	6.67 ± 0.33$^{b, A}$
2 - PMA	-	4.77 ± 0.52$^{a, AB}$	5.80 ± 0.71$^{a, A}$	6.33 ± 0.1$^{a, A}$	5.87 ± 0.3$^{a, AB}$	6.70 ± 0.25$^{a, B}$	5.70 ± 0.23$^{a, A}$
3 - PMA	-	4.37 ± 0.62$^{a, AB}$	6.50 ± 0.33$^{bc, A}$	5.68 ± 0.26$^{ac, AB}$	7.00 ± 0.26$^{bc, A}$	7.58 ± 0.13$^{b, A}$	6.60 ± 0.15$^{ac, A}$
4 - PMA	-	5.60 ± 0.26$^{a, A}$	6.92 ± 0.09$^{b, A}$	6.10 ± 0.08$^{ab, AB}$	6.90 ± 0.25$^{b, A}$	6.60 ± 0.13$^{b, B}$	6.25 ± 0.24$^{ab, A}$
5 - PMA	-	4.22 ± 0.32$^{a, B}$	6.77 ± 0.23$^{c, A}$	5.32 ± 0.25$^{b, B}$	5.40 ± 0.27$^{b, B}$	6.74 ± 0.07$^{c, B}$	6.12 ± 0.19$^{bc, A}$

d.

	Age (months)						
**Trial number**	**0-1**	**3-6**	**7-10**	**13-15**	**18-20**	**24**	**30-32**
1	3.91 ± 0.15^{a}	4.72 ± 0.34$^{abc, B}$	5.64 ± 0.24$^{c, AB}$	5.44 ± 0.08$^{bc, A}$	4.06 ± 0.25$^{ab, A}$	4.86 ± 0.29$^{abc, A}$	4.18 ± 0.42$^{ab, AB}$
2	3.43 ± 0.02^{a}	4.40 ± 0.29$^{a, B}$	4.78 ± 0.21$^{bc, B}$	5.20 ± 0.06$^{bc, AB}$	4.94 ± 0.21$^{bc, B}$	5.63 ± 0.16$^{a, AB}$	3.12 ± 0.14$^{a, A}$
3	BDL	3.06 ± 0.19$^{a, A}$	4.57 ± 0.47$^{b, AB}$	4.49 ± 0.17$^{b, BC}$	5.45 ± 0.24$^{bc, B}$	5.64 ± 0.13$^{c, AB}$	5.26 ± 0.20$^{bc, BC}$
4	BDL	7.78 ± 0.19$^{c, C}$	7.64 ± 0.17$^{c, AB}$	2.87 ± 0.39$^{a, C}$	8.12 ± 0.05$^{c, C}$	5.35 ± 0.25$^{b, AB}$	4.55 ± 0.18$^{b, BC}$
5	BDL	3.76 ± 0.28$^{a, AB}$	5.37 ± 0.43$^{ab, C}$	3.54 ± 0.30$^{a, C}$	4.69 ± 0.18$^{ac, AB}$	5.67 ± 0.06$^{b, C}$	5.34 ± 0.25$^{bc, C}$
1 - PMA	-	3.91 ± 0.36$^{a, A}$	5.73 ± 0.24$^{b, A}$	5.07 ± 0.29$^{ab, AB}$	4.35 ± 0.14$^{ab, A}$	4.84 ± 0.30$^{ab, AB}$	4.14 ± 0.32$^{a, AB}$
2 - PMA	-	3.71 ± 0.4$^{abc, AB}$	4.60 ± 0.34$^{abc, A}$	5.17 ± 0.05$^{ab, A}$	4.77 ± 0.23$^{ab, A}$	5.93 ± 0.29$^{b, AB}$	3.05 ± 0.12$^{c, A}$
3 - PMA	-	2.13 ± 0.13$^{a, B}$	2.06 ± 0.36$^{a, B}$	3.98 ± 0.36$^{b, BC}$	4.87 ± 0.35$^{b, A}$	4.63 ± 0.14$^{b, A}$	3.86 ± 0.44$^{b, AB}$
4 - PMA	-	7.11 ± 0.21$^{b, C}$	8.46 ± 0.12$^{a, C}$	3.80 ± 0.22$^{d, C}$	8.15 ± 0.20$^{a, B}$	5.08 ± 0.26$^{c, AB}$	4.32 ± 0.25$^{cd, B}$
5 - PMA	-	3.22 ± 0.26$^{a, A}$	5.47 ± 0.24$^{a, A}$	4.04 ± 0.27$^{ac, ABC}$	4.05 ± 0.15$^{ac, A}$	5.58 ± 0.03$^{b, B}$	4.99 ± 0.22$^{bc, B}$

* Different lower case letters (a-d) indicate significant differences between age groups within the same trial (p ≦ 0.05). In section a. of the table, different capital letters (A and B) indicate significant differences between total and PMA-treated DNA within the same age group except for 0—1 m (p ≦ 0.05) identified by the Independent Samples *t* Test. In sections b, c and d, different capital letters (A-C) indicate significant differences between trials within the same age group except for 0—1 m in section d (p ≦ 0.05) over trials. The statistical comparisons were carried out using Tukey's Honestly Significant Difference test (where there were no violations of the test assumptions for the homogeneity of variance, Levene's k - sample comparison of variances or 1 - way ANOVA using the Games - Howell post-hoc analysis where the test assumptions for the homogeneity of variance were violated). Data are the mean values ± standard error from at least three separate samples. BDL: < 2 log.

Table S3. Average relative abundance of *Lactococcus* spp. and NSLAB using 16S rRNA gene amplicon sequencing (% of total reads identified to genus level). Sampling was performed over 32 m at 0-1 m (11 trials for total DNA, 4 trials for PMA-treated DNA), 3-6 m (13 trials), 7-10 m (13 trials), 13-15 m (13 trials), 18-20 m (11 trials), 24 m (7 trials) and 30-32 m (5 trials) time points. .

	Age (month)						
**Group of taxa**	**0-1 m (total n=11, PMA n=4) ‡**	**3-6 m (n=13)**	**7-10 (n=13)**	**13-15 (n=13)**	**18-20 m (n=11)**	**24 m (n=7)**	**30-32 (n=5)**
Lactococcus spp. Total	97.85 ± 0.89^{a,A}	60.47 ± 5.1^{b,A}	26.83 ± 6.75^{c,A}	12.07 ± 2.55^{d,A}	14.72 ± 4.67^{cde,A}	2.90 ± 1.37^{ef,A}	4.78 ± 2.97^{f,A}
Lactococcus spp. Viable	97.05 ± 0.77^{a,A}	24.62 ± 5.07^{b,B}	7.80 ± 3.16^{c,B}	2.11 ± 1d$^{e, B}$	4.06 ± 1.67^{de,B}	4.44 ± 3.51^{de,A}	0.15 ± 0.05^{e,A}
NSLAB Total	1.95 ± 0.89^{a,A}	38.03 ± 5.05^{b,A}	71.67 ± 6.78^{c,A}	84.55 ± 2.87^{d,A}	84.37 ± 4.75^{cde,A}	94.69 ± 1.8^{e,A}	84.19 ± 4.2d^{e,A}
NSLAB Viable	2.92 ± 0.76^{a,A}	72.48 ± 4.97^{b,B}	91.25 ± 3.43^{c,B}	92.89 ± 2.12^{c,B}	92.91 ± 2.43^{c,B}	94.38 ± 3.45^{c,A}	90.54 ± 1.72^{c,A}

* Different lower case letters (a-f) indicate significant differences between age groups within the same trial (p ≦ 0.05) The statistical comparisons were carried out using Tukey's Honestly Significant Difference test (where there were no violations of the test assumptions for the homogeneity of variance, Levene's k - sample comparison of variances or 1 - way ANOVA using the Games - Howell post-hoc analysis where the test assumptions for the homogeneity of variance were violated). Different capital letters (A and B) indicate significant differences between total and PMA-treated DNA within the same age group (p ≦ 0.05) identified by "Independent Samples *t* Test". ‡ Cell pellets obtained from cheeses at 0-1 m were not treated with PMA for trials 1-7, 16S rRNA gene amplicon sequencing data are missing for cheeses from trials 12 and 13 at 0-1 m.

Table S4. Average relative abundance of *Lactococcus* spp. using 16S rRNA gene amplicon sequencing per trial (% of total reads identified to genus level) over a maximum of 32 months of ripening. a. total and b. Sampling was performed over a course of 32 m at 0-1 m (11 trials for total DNA, 4 trials for PMA-treated DNA), 3-6 m (13 trials), 7-10 m (13 trials), 13-15 m (13 trials), 18-20 m (11 trials), 24 m (7 trials) and 30-32 m (5 trials) time points.

a.

	Age (months)						
**Trial number**	**0-1**	**4-6**	**7-10**	**13-15**	**18-20**	**24**	**30-32**
1	93.81 ± 2.1$^{a, AB*}$	69.04 ± 9.27 $^{a, ABC}$	33.76 ± 14.90 $^{ab, BCDE}$	7.34 ± 3.19$^{b, AB}$	4.80 ± 0.59 $^{b, A}$	3.16 ± 0.53 $^{b, A}$	1.07 ± 0.12 $^{b, A}$
2	94.73 ± 4.26 $^{a, ABC}$	69.53 ± 8.22 $^{ab, ABC}$	42.61 ± 6.47 $^{bd, BEF}$	3.05 ± 0.98 $^{c, A}$	9.01 ± 3.57 $^{cd, AB}$	1.50 ± 0.27 $^{c, A}$	1.37 ± 0.27 $^{c, A}$
3	99.04 ± 0.64 $^{a, BC}$	73.04 ± 6.56 $^{ab, BC}$	73.74 ± 4.61 $^{b, F}$	30.06 ± 9.32 $^{bc, B}$	44.34 ± 10.57$^{abc, B}$	0.52 ± 0.18 $^{c, A}$	16.59 ± 4.05 $^{c, B}$
4	99.34 ± 0.59 $^{a, BC}$	73.26 ± 7.00 $^{a, ABC}$	11.31 ± 2.44 $^{b, ABCD}$	4.87 ± 1.15 $^{b, AB}$	5.51 ± 2.71 $^{b, A}$	1.24 ± 0.39 $^{b, A}$	2.85 ± 0.67 $^{b, B}$
5	99.59 ± 0.09 $^{a, BC}$	91.53 ± 3.88 $^{a, C}$	62.00 ± 8.72 $^{ac, EF}$	26.44 ± 8.67 $^{bc, AB}$	35.46 ± 13.85$^{abc, AB}$	8.10 ± 7.09 $^{b, A}$	2.00 ± 0.35 $^{b, B}$
6	100.0 ± 0.00 $^{a, BC}$	39.26 ± 10.95 $^{b, AB}$	1.50 ± 0.35 $^{c, B}$	3.09 ± 0.99 $^{c, AB}$	33.99 ± 11.82$^{b, AB}$	0.74 ± 0.23 $^{c, A}$	-
7	99.63 ± 0.28 $^{a, BC}$	66.58 ± 8.77 $^{b, ABC}$	8.59 ± 2.75 $^{c, ABC}$	14.50 ± 7.17 $^{c, AB}$	0.04 ± 0.02 $^{c, A}$	1.65 ± 0.39 $^{c, A}$	-
8	99.82 ± 0.02 $^{a, C}$	79.21 ± 6.15 $^{b, BC}$	12.65 ± 4.86 $^{c, ABCD}$	4.74 ± 1.91 $^{c, AB}$	12.55 ± 2.28 $^{c, AB}$	-	-
9	99.94 ± 0.04 $^{a, C}$	33.45 ± 9.78 $^{b, A}$	11.87 ± 1.25 $^{c, ABCD,}$	19.13 ± 3.12 $^{ab, AB}$	2.16 ± 2 $^{c, A}$	-	-
10	98.72 ± 0.90 $^{a, BC}$	61.61 ± 15.33 $^{ab, ABC}$	36.98 ± 13.36 $^{bc, CDE}$	14.00 ± 5.37 $^{c, AB}$	8.28 ± 1.73 $^{c, AB}$	-	-
11	91.73 ± 1.82 $^{a, A}$	33.83 ± 7.93 $^{bc, A}$	48.28 ± 10.48 $^{b, EF}$	13.33 ± 2.74 $^{cd, AB}$	5.75 ± 1.05 $^{d, A}$	-	-
12	-	50.81 ± 12.60 $^{a, ABC}$	0.07 ± 0.02 $^{b, A}$	15.45 ± 7.69 $^{b, AB}$	-	-	-
13	-	45.00 ± 8.82 $^{a, AB}$	5.45 ± 1.46 $^{b, ABC}$	0.90 ± 0.15 $^{b, A}$	-	-	-

b.

	Age (months)						
**Trial number**	**0-1‡**	**3-6**	**7-10**	**13-15**	**18-20**	**24**	**30-32**
1	-	30.84 ± 9.07$^{bc, AB}$	7.98 ± 3.2 $^{bc, A}$	0.37 ± 0.07 $^{bc, A}$	0.44 ± 0.04 $^{b, A}$	0.35 ± 0.06 $^{bc, A}$	0.03 ± 0.03 $^{c, A}$
2	-	19.60 ± 6.98 $^{a, AB}$	1.79 ± 0.45 $^{a, A}$	0.76 ± 0.34 $^{b, A}$	0.24 ± 0.09 $^{b, A}$	2.36 ± 0.36 $^{b, A}$	0.10 ± 0.06 $^{b, A}$
3	-	37.75 ± 10.71$^{bc, AB}$	38.71 ± 8.56 $^{b, B}$	4.34 ± 3.07 $^{bc, A}$	3.32 ± 2.75 $^{bc, A}$	0.88 ± 0.66 $^{bc, A}$	0.30 ± 0.04 $^{c, A}$
4	-	21.34 ± 7.4 $^{b, AB}$	0.98 ± 0.45 $^{b, A}$	0.08 ± 0.04 $^{b, A}$	0.11 ± 0.05 $^{b, A}$	0.24 ± 0.22 $^{b, A}$	0.15 ± 0.03 $^{b, A}$
5	-	51.44 ± 11.22 $^{b, B}$	17.25 ± 6.36 $^{b, A}$	4.88 ± 2.34 $^{b, A}$	1.64 ± 0.6 $^{b, A}$	18.39 ± 10.56 $^{b, A}$	0.19 ± 0.11 $^{b, A}$
6	-	0.13 ± 0.03$^{a, A}$	0.23 ± 0.05 $^{a, A}$	0.02 ± 0 $^{a, A}$	9.78 ± 5.49 $^{a, A}$	0.11 ± 0.08 $^{a, A}$	-
7	-	22.32 ± 7.58 $^{b, AB}$	0.31 ± 0.09 $^{a, A}$	0.75 ± 0.23 $^{a, A}$	1.20 ± 0.23 $^{a, A}$	0.01 ± 0.01 $^{a, A}$	-
8	99.68 ± 0.06 $^{a, A}$	58.02 ± 9.04 $^{b, B}$	2.54 ± 1.5 $^{c, A}$	0.06 ± 0.03 $^{c, A}$	10.85 ± 0.92 $^{c, A}$	-	-
9	99.89 ± 0.07 $^{a, A}$	4.34 ± 3.14 $^{b, A}$	2.04 ± 1.03 $^{b, A}$	1.97 ± 0.51 $^{b, A}$	16.08 ± 8.95 $^{b, A}$	-	-
10	99.90 ± 0.04 $^{a, A}$	34.68 ± 15.28 $^{b, AB}$	20.52 ± 10.12 $^{b, AB}$	0.80 ± 0.49 $^{c, A}$	0.52 ± 0.12 $^{c, A}$	-	-
11	88.71 ± 2.89 $^{a, A}$	5.31 ± 1.73 $^{b, A}$	7.02 ± 2.23 $^{b, A}$	0.50 ± 0.22 $^{b, A}$	0.45 ± 0.10 $^{b, A}$	-	-
12	-	29.85 ± 12.12 $^{a, AB}$	1.51 ± 0.48 $^{b, A}$	12.83 ± 8.11 $^{b, A}$	-	-	-
13	-	4.46 ± 1.47 $^{a, A}$	0.53 ± 0.28 $^{b, A}$	0.13 ± 0.06 $^{b, A}$	-	-	-

* Different lower case letters (a-d) indicate significant differences between age groups within the same trial ($p \leqq 0.05$) and different capital letters (A-F) indicate significant differences between trials within the same age group ($p \leqq 0.05$) over trials. The statistical comparisons were carried out using Tukey's Honestly Significant Difference test (where there were no violations of the test assumptions for the homogeneity of variance, Levene's k - sample comparison of variances or 1 - way ANOVA using the Games - Howell post-hoc analysis where the test assumptions for the homogeneity of variance were violated). Data are the mean values ± standard error from at least three separate samples. ‡ Cheeses at 0-1 m were not treated with PMA for samples from trials 1-7, while 16S rRNA gene amplicon sequencing data are missing for cheeses from trials 12 and 13 at 0-1 m.

Table S5. Average relative abundance of NSLAB using 16S rRNA gene amplicon sequencing per trial (% of total reads identified to genus level per trial) over a maximum of 32 months of ripening. a. total DNA and b. DNA from PMA-treated cells. Sampling was performed over a course of 32 m at 0-1 m (11 trials for total DNA, 4 trials for PMA-treated DNA), 3-6 m (13 trials), 7-10 m (13 trials), 13-15 m (13 trials), 18-20 m (11 trials), 24 m (7 trials) and 30-32 m (5 trials) time points.

a.

Trial number	Age (months) 0-1	4-6	7-10	13-15	18-20	24	30-32
1	5.81 ± 1.97 [a, BC*]	29.65 ± 8.88 [a, ABC]	65.82 ± 14.78 [abc, BCDEF]	91.69 ± 3.15 [bc, AB]	93.80 ± 0.82 [b, B]	89.97 ± 0.95 [bc, A]	85.70 ± 1.15 [c, A]
2	5.08 ± 4.16 [a, ABC]	28.95 ± 8.18 [ab, ABC]	53.86 ± 7.19 [bd, ABCD]	96.25 ± 1.11 [c, B]	90.99 ± 3.57 [cd, B]	98.05 ± 0.34 [c, A]	88.70 ± 0.44 [d, A]
3	0.90 ± 0.6 [a, AB]	24.01 ± 7.05 [ab, ABC]	23.93 ± 5.13 [b, A]	64.58 ± 8.56 [bc, A]	53.09 ± 10.09 [ac, A]	95.76 ± 1.02 [c, A]	67.92 ± 3.6 [c, B]
4	0.66 ± 0.59 [a, AB]	26.74 ± 7.00 [a, ABC]	88.69 ± 2.44 [bc, EFG]	93.60 ± 1.29 [bc, B]	94.27 ± 2.76 [bc, B]	98.69 ± 0.38 [b, A]	86.80 ± 1.99 [c, B]
5	0.24 ± 0.05 [a, A]	7.93 ± 3.87 [a, A]	37.32 ± 8.54 [ab, AB]	70.75 ± 8.44 [bc, AB]	64.20 ± 13.8 [ac, AB]	90.98 ± 6.86 [c, A]	91.83 ± 1.14 [c, B]
6	0.01 ± 0.00 [a, A]	57.31 ± 10.35 [b, BC]	96.84 ± 0.35 [d, FG]	91.92 ± 1.91 [cd, B]	65.40 ± 11.76 [bc, AB]	97.43 ± 1.33 [d, A]	-
7	0.34 ± 0.29 [a, AB]	32.43 ± 8.93 [b, ABC]	89.92 ± 2.67 [c, EFG]	81.18 ± 7.63 [c, AB]	99.37 ± 0.30 [c, B]	86.41 ± 2.02 [c, A]	-
8	0.12 ± 0.01 [a, A]	18.83 ± 5.62 [a, AB]	84.88 ± 5.05 [b, DEFG]	93.85 ± 1.89 [b, B]	85.58 ± 2.87 [b, AB]	-	-
9	0.03 ± 0.02 [a, A]	66.16 ± 9.89 [b, C]	82.85 ± 2.03 [bc, CDEFG]	77.94 ± 3.29 [bc, AB]	96.08 ± 2.33 [c, B]	-	-
10	0.04 ± 0.01 [a, A]	36.88 ± 14.94 [ab, ABC]	62.79 ± 13.38 [bc, BCDE]	85.00 ± 4.73 [c, AB]	91.36 ± 1.72 [c, B]	-	-
11	8.22 ± 1.81 [a, C]	64.60 ± 7.89 [b, C]	50.84 ± 10.58 [b, ABC]	74.33 ± 4.43 [bc, AB]	93.90 ± 1.02 [c, B]	-	-
12	-	47.35 ± 12.97 [a, ABC]	99.73 ± 0.04 [b, G]	82.42 ± 7.48 [b, AB]	-	-	-
13	-	53.60 ± 9.15 [a, BC]	94.20 ± 1.49 [b, EFG]	95.70 ± 0.62 [b, B]	-	-	-

b.

Trial number	Age (months) 0-1‡	3-6	7-10	13-15	18-20	24	30-32
1	-	66.10 ± 9.21 [a, ABC]	91.76 ± 3.22 [a, B]	99.06 ± 0.33 [a, B]	98.77 ± 0.32 [a, A]	97.07 ± 0.71 [a, A]	91.47 ± 1.47 [a, AB]
2	-	79.21 ± 7.55 [ab, ABC]	97.10 ± 0.84 [a, B]	98.97 ± 0.44 [a, B]	99.76 ± 0.09 [a, A]	97.49 ± 0.36 [a, A]	91.07 ± 0.55 [b, AB]
3	-	56.09 ± 13.11 [abc, ABC]	56.62 ± 11.03 [bc, A]	91.78 ± 3.3 [bc, B]	90.23 ± 3.24 [bc, A]	97.16 ± 1.11 [b, A]	83.92 ± 1.61 [c, A]
4	-	76.35 ± 7.57 [a, ABC]	99.02 ± 0.44 [a, B]	99.29 ± 0.41 [a, B]	99.72 ± 0.05 [a, A]	99.49 ± 0.3 [a, A]	92.48 ± 2.60 [a, B]
5	-	47.52 ± 11.06 [a, AB]	82.27 ± 6.34 [a, B]	93.29 ± 2.37 [a, B]	76.18 ± 9.34 [a, A]	80.69 ± 10.28 [a, A]	93.74 ± 1.62 [a, B]
6	-	99.74 ± 0.05 [a, C]	99.58 ± 0.07 [a, B]	86.95 ± 5.47 [a, AB]	89.05 ± 5.9 [a, A]	96.78 ± 1.49 [a, A]	-
7	-	76.79 ± 7.99 [a, ABC]	99.21 ± 0.23 [b, B]	96.44 ± 0.9 [b, B]	98.33 ± 0.27 [b, A]	86.39 ± 2.60 [ab, A]	-
8	0.32 ± 0.06 [a, A]	39.33 ± 8.52 [b, A]	96.61 ± 1.69 [c, B]	99.56 ± 0.06 [c, B]	88.19 ± 0.88 [c, A]	-	-
9	0.06 ± 0.05 [a, A]	89.25 ± 6.97 [bc, BC]	97.04 ± 1.25 [c, B]	73.63 ± 4.58 [b, A]	83.41 ± 8.84 [bc, A]	-	-
10	0.03 ± 0.02 [a, A]	64.28 ± 15.22 [b, ABC]	78.93 ± 10.21 [bc, AB]	97.40 ± 0.93 [bc, B]	99.22 ± 0.16 [c, A]	-	-
11	11.28 ± 2.89 [a, A]	84.43 ± 4.2 [b, BC]	90.74 ± 2.35 [bc, B]	88.50 ± 0.34 [bc, AB]	99.20 ± 0.07 [c, A]	-	-
12	-	68.40 ± 12.67 [a, ABC]	98.07 ± 0.57 [a, B]	85.18 ± 7.91 [a, AB]	-	-	-
13	-	94.79 ± 1.73 [a, C]	99.32 ± 0.25 [ab, B]	97.52 ± 0.60 [b, B]	-	-	-

* Different lower case letters (a-d) indicate significant differences between age groups within the same trial (p ≦ 0.05) and different capital letters (A-G) indicate significant differences between trials within the same age group (p ≦ 0.05). The statistical comparisons were carried out using Tukey's Honestly Significant Difference test (where there were no violations of the test assumptions for the homogeneity of variance, Levene's k - sample comparison of variances or 1 - way ANOVA using the Games - Howell post-hoc analysis where the test assumptions for the homogeneity of variance were violated). Data are the mean values ± standard error from at least three separate samples. Data are the mean values ± standard error from at least three separate samples. ‡ Cheeses at 0-1 m were not treated with PMA for samples from trials 1-7, while 16S rRNA gene amplicon sequencing data are missing for trials 12 and 13 at 0-1 m.

Table S6. Number of ASVs, a. Non-filtered dataset (a total of 3,491 ASVs) and b. Filtered dataset at 0.1% abundance in over 10% of the samples (a total of 742 ASVs).

a.

Taxonomy	Number of ASVs
Lactococcus	133
Lacticaseibacillus	520
Latilactobacillus	133
Paucilactobacillus	126
Pediococcus	88
Secundilactobacillus	120
Streptococcus	198
Weissella	233
Enterobacter	165
Other taxa	1775

b.

Taxonomy	Number of ASVs
Lactococcus	55
Lacticaseibacillus	182
Latilactobacillus	76
Paucilactobacillus	54
Pediococcus	51
Secundilactobacillus	81
Streptococcus	53
Weissella	97
Enterobacter	93

Table S7. Total number of ASVs for *Lactococcus* spp. and *Lacticaseibacillus* spp. across 13 trials.

Taxonomic Group	Total	Average	SD
Lactococcus spp.	133	100	±13.9
Lacticaseibacillus spp.	520	378	±64.6

Table S8. Number of ASVs for *Lactococcus* spp. and *Lacticaseibacillus* spp. for each of 13 trials.

Trial number	*Lactococcus*	*Lacticaseibacillus*
1	86	297
2	101	391
3	109	433
4	107	432
5	112	462
6	118	443
7	116	436
8	96	354
9	99	391
10	81	285
11	90	297
12	111	396
13	74	297
